# Single-cell RNA-seq uncovered hemocyte functional subtypes and their differentiational characteristics and connectivity with morphological subpopulations in *Litopenaeus vannamei*

**DOI:** 10.3389/fimmu.2022.980021

**Published:** 2022-09-13

**Authors:** Chuang Cui, Xiaoqian Tang, Jing Xing, Xiuzhen Sheng, Heng Chi, Wenbin Zhan

**Affiliations:** ^1^ Laboratory of Pathology and Immunology of Aquatic Animals, The Key Laboratory of Mariculture, Ministry of Education (KLMME), Ocean University of China, Qingdao, China; ^2^ Laboratory for Marine Fisheries Science and Food Production Processes, Qingdao National Laboratory for Marine Science and Technology, Qingdao, China

**Keywords:** single-cell RNA-seq, hemocytes, *Litopenaeus vannamei*, functional cluster, differentiation trajectory

## Abstract

Hemocytes play central roles in shrimp immune system, whereas whose subclasses have not yet been completely defined. At present, the morphological classification of hemocytes is inadequate to classify the complete hemocyte repertoire and elucidate the functions and differentiation and maturation processes. Based on single-cell RNA sequencing (scRNA-seq) of hemocytes in healthy *Litopenaeus vannamei*, combined with RNA-FISH and flow cytometric sorting, we identified three hemocyte clusters including TGase^+^ cells, CTL^+^ cells and Crustin^+^ cells, and further determined their functional properties, potential differentiation trajectory and correspondence with morphological subpopulations. The TGase^+^ cells were mainly responsible for the coagulation, exhibiting distinguishable characteristics of hyalinocyte, and appeared to be developmentally arrested at an early stage of hemocyte differentiation. The CTL^+^ cells and Crustin^+^ cells arrested at terminal stages of differentiation mainly participated in recognizing foreign pathogens and initiating immune defense responses, owning distinctive features of granule-containing hemocytes. Furthermore, we have revealed the functional sub-clusters of three hemocyte clusters and their potential differentiation pathways according to the expression of genes involved in cell cycle, cell differentiation and immune response, and the successive differentiation and maturation of hyalinocytes to granule-containing hemocytes have also mapped. The results revealed the diversity of shrimp hemocytes and provide new theoretical rationale for hemocyte classification, which also facilitate systematic research on crustacean immunity.

## Introduction

Shrimp have been considered to the most heavily traded fish products and shrimp culture is one of the fastest growing industries for producing animal proteins in the world (1, 2). The *Litopenaeus vannamei* is the most important cultured shrimp worldwide, accounting for approximately 80% of total cultured shrimp production (3). Over the past decades, disease still represent a major impediment to the development of shrimp culture, and disease outbreaks result in large economic losses to the industry (4). As a typical aquatic invertebrate, shrimps rely on innate immune system for defense against pathogenic infection (5, 6), in which hemocytes perform central roles *via* multiple mechanisms. The hemocytes participate in humoral immune responses by synthesis and release of antimicrobial peptides, lectins, reactive oxygen species and inflammatory cytokines, and also are involved in cellular immunity *via* recognition of foreign pathogens, phagocytosis, melanization and apoptosis (7–10). Therefore, it is vital to characterize hemocytes systemically and comprehensively for deepening understanding of their immune roles in shrimp, which would benefit designing novel and efficient strategies for disease prevention and control.

Due to the lack of marker genes available to define cell types, the classification of crustacean hemocytes is still performed on the basis of morphological characteristics (11–13). In shrimp, hemocytes are also classified into granulocytes, semi-granulocytes and hyalinocytes depending on their morphology and intracellular granule staining characteristics (9, 14). However, the functional properties and differentiation relationships of the three hemocyte subpopulations have not been fully revealed. Moreover, there is inconsistent finding regarding hemocytes classification, some researches have classified shrimp hemocytes into four types by ultrastructural and microscopic observation (15), five types by iodixanol density gradient centrifugation (16), two or three types by lectin staining (17, 18), and even much more types based on differential binding affinities of hemocytes towards monoclonal antibodies (19, 20). Nevertheless, none of these cell subpopulations have been characterized at the molecular level and their transcriptional profiles remain unknown, and the correspondence between subpopulations and differentiation stages are still also ambiguous and vague (21, 22). In addition, the classification based on low-resolution of morphological and staining characteristics is difficult to identify rare cell types and might hinder distinguishing the cells in transient state (9, 23, 24). Hence, it is important to thoroughly unravel molecular signatures of hemocytes, which enable elucidation of functional and differentiation characteristics of hemocytes subpopulation and efficient identification of marker genes.

In recent years, the emergence and advancement of single-cell RNA sequencing (scRNA-seq) technologies has made it possible to comprehensively characterize complex tissues cells at the molecular level (25, 26). The scRNA-seq provides access to the dynamic gene expression patterns of single cells and according to the large amount of transcript information obtained, it is possible to accurately characterize cell type, annotate the unclassified cells, explore marker genes and map the developmental trajectories of cellular lineages, thus providing a thorough analysis of cell heterogeneity and their states (27–29). Single cell transcriptome technology has been widely used in the research on the properties of the organ and tissue cells in plants and animals (30–32). Meanwhile, the successful applications of scRNA-seq on invertebrate hemocytes from *Drosophila*, mosquitoes and oysters have established the basis for scRNA-seq studies on shrimp and further revealed the significance of characterizing the functional properties of hemocytes at the single-cell transcriptional level (33–35).

Here, we performed scRNA-seq of hemocytes from healthy *L. vannamei* to distinguish the hemocytes types and explore their functional properties. As a result, thousands of hemocytes transcripts were obtained, which were utilized to identify the main cell types and their putative marker genes, characterize their function and reveal the differentiation relationships among different cell types. Moreover, we sorted morphological subpopulations of hemocytes using flow cytometry and organically aligned them with the above cell types. In summary, our scRNA-seq analysis revealed the diversity of the shrimp hemocyte populations and provided a deeper understanding of the hemocytes subpopulations.

## Materials and methods

### Shrimp and hemocytes preparation

The healthy male adult shrimp (*Litopenaeus vannamei*) of 30-40 g were obtained from an aquaculture farm in Qingdao, Shandong Province. The shrimp were temporarily maintained at 23°C in a recirculating system containing aerated filtered seawater for one week and then used for experimental studies. The studies were carried out in agreement with the International Guiding Principles for Biomedical Research Involving Animals documented by Guide for the Use of Experimental Animals and the Committee of the Ethics on Animal Care and Experiments at Ocean University of China (permit number: 20180101).

Using sterile modified cold Alsever solution (27 mM Na citrate, 336 mM NaCl, 115 mM glucose, 9 mM EDTA, pH 7.2, AS) as anticoagulant, hemolymph was withdrawn from the pericardial cavity of three randomly selected healthy prawns and pooled in a sterile centrifuge tube (7). The hemocytes suspension was then centrifuged at 400g for 5 min at 4°C and washed three times with sterile prawn homoiosmotic phosphate buffered saline (377 mM NaCl, 2.70 mM KCl, 8.09 mM Na_2_HPO_4_, 1.47 mM KH_2_PO_4_, pH 7.4, 780 mOsm/L, PHPBS). After washing, the prepared hemocytes suspension was passed through a 40 μm cell strainer (Corning, USA) to remove cell aggregates and counted on a cell counting plate. Cellular viability was measured using a Trypan Blue Staining kit (Solarbio) according to the operating instructions.

### Single hemocyte encapsulation and sequencing

Single cells encapsulation, library synthesis and RNA sequencing were performed by the Gene Denovo (Guangzhou, China). In brief, the concentration of hemocytes was adjusted to 1500–2000 cells/μl. Each single cell was encapsulated in a droplet to generate a Gel Beads-In-Emulsions (GEMs) using 10X Genomics GemCode Technology according to the manufacturer’s instructions. Every cell was barcode-labeled, and every transcript was labeled with a unique molecular identifier (UMI). The cDNA library was then amplified by PCR with the sequencing primer R1 and P5 arm. The concentration of cDNA library was measured by Qubit (Invitrogen) and qualitative analysis of cDNA library was performed by an Agilent 2100 Bioanalyzer. The qualified cDNA libraries were sequenced on the Illumina 10X Genomics Chromium platform.

### Quality control and data processing

The obtained raw single-cell RNA sequencing dataset was submitted for data quality statistics, reads aligning and generating gene-cell matrices by the official 10X Genomics analysis software Cell Ranger. Read1 and Read2 were obtained by Illumina double-end sequencing, with Read1 containing a 16 bp size Gemcode for distinguishing different cells and a 10 bp size UMI for labeling each transcript, while Read2 contained mainly cDNA sequence fragments. The Spliced Transcripts Alignment to a Reference alignment software (STAR) of Cell Ranger was used to compare Read2 with the reference genome of *L. vannamei* (ASM378908v1) (36, 37). The reads located in exons were aligned with transcripts containing annotation information, and if the two were in the same direction, they were considered to be transcriptome and annotated as transcriptome reads. In transcriptome reads, if the reads matched only one gene, it was considered to be un-mapped. UMI counting was only available using uni-mapped reads. Subsequently, quality control analysis was performed to filter and correct for barcodes and UMIs. The QC relies on the following criteria: (1) 500 < gene counts < 4000 per cell. (2) UMI counts < 8000 per cell. (3) the percentage of mitochondrial genes < 10%. UMIs were not allowed to be single oligo-chains and were not able to contain bases with a mass value of less than 10. Meanwhile, UMI counting could only be conducted by valid comments with validate barcode and UMI. Following removal of the UMI repeats, the number of unique UMIs was calculated as the expression level of the cellular gene. The filtered and quantified raw data was generated by Cell Ranger as gene-cell matrices and used for further analysis.

### Data dimensionality reduction and cell clustering

The eligible gene-cell matrices were loaded into the Seurat package of R software for cell clustering and dimensionality reduction analysis. The data were first normalized by ‘LogNormalize’ function and the variations between cells were regressed out by counting the number of target molecules in each cell (UMI). Then, Principal Component Analysis (PCA) was performed on the scaled data for dimensionality reduction, and the low *P-*values genes were strong enrichment. The distribution of *P*-values was compared by the JackStrawPlot function, and the most significant principal component (PC, *p <*10-5) was selected in the PCA results for downstream clustering and cluster analysis (38). Based on the previously determined PCs, the cells were clustered and grouped using the ‘FindClusters’ function. The data structures and cell trajectories were separately visualized and explored by *t*-SNE and UMAP (39, 40).

### Marker gene identification

For the cell clusters obtained above, marker genes (cluster-enriched genes) were identified and tested by Seurat function ‘FindAllMarkers’ and ‘roc’, respectively. Following this analysis, cluster-specific marker genes were further screened for genes expressed in more than 50% cells of a cluster and an average natural log-fold change greater than 0.5. Multiple replicate analyses were performed using the same or different parameters to verify the accuracy of the marker genes.

### Differential expression genes analysis

To further characterize the transcriptional regulatory patterns of each hemocyte cluster, likelihood ratio-tests were used to find the DEGs of individual cell clusters compared to all other hemocyte clusters (41). Subsequently, differential gene expression analysis was performed on different cell populations using the Seurat’bimod’ function. The up-regulated genes in each hemocyte cluster were screened for functional analysis based on the conditions |logFC| ≥ 0.25, *p*-value ≤ 0.05, and the percentages of cells in specific cluster for which the gene was detected to be > 25%. Based on the differentially upregulated genes expression profiles in each of the cell clusters, we first used gene ontology (GO) enrichment to analyze the biological function in each cell type and the important differentially expressed transcripts (42). The differentially expressed transcript was mapped to GO terms in Gene Ontology database (http://www.geneontology.org/), and a list of transcripts and transcriptions with a certain GO functions were obtained by calculating the number of genes per term. Then, hypergeometric tests were applied to define GO terms that were significantly enriched in peak-associated genes relative to the genomic background and to identify significant biological functions of differentially expressed transcripts. Meanwhile, KEGG pathway analysis was used to unravel the coordinated interactions between different transcripts and obtain the enriched pathways in different cell clusters (43). Using the KEGG pathway as a unit, the pathways significantly enriched in differentially expressed transcripts were extracted by hypergeometric testing to further analyze the biological functions of differentially transcribed genes in each cluster.

### Pseudotemporal ordering of cells using Monocle 3

The Monocle (v.3.0) package was utilized for in-depth analysis of the cell differentiation process and cell fate. Based on the previously identified cell clusters and differentially expressed genes, the above data were transferred to Monocle package, then the differentiation and cell fate related genes were selected to define the cell differentiation process. Monocle compressed the data dimensionality to one with two dimensions and arranges all cells in an ordered manner. Genes with similar expression trends and might share common biological functions were grouped together to calculate cell trajectories using the ‘learning-graph’ function.

### Sub-clustering analysis

We applied the ‘FindClusters’ function to each of the obtained cell clusters to perform sub-cluster analysis with a view to clarifying the subpopulation composition and functional relationships of the cell clusters. The transcript expression patterns of each secondary subpopulation were clarified using gene differential analysis, and all sub-clusters obtained were reconstructed by Monocle3 package for the Pseudotime analysis.

### Fluorescent *in situ* hybridization

The FISH procedure was carried out strictly according to the manufacturer’s protocol of FISH probes kit (GenePharam). Three healthy shrimp hemocytes were drawn, washed and fixed on slides with 4% paraformaldehyde. The samples were sequentially dehydrated with 100%, 95%, 85% and 75% ethanol for 2 min. After pepsin digestion for 5 min, the samples were washed with hybridization solution. The nucleic acid probes with fluorescent labels were then incubated overnight at 37°C. The sensing probe was used as a negative control. Following washing, the samples were observed by fluorescence microscopy. The nucleic acid probes used for the experiments were synthesized by GenePharam and Tsingke (seen **Table 1**).

**Table 1:** Probes used in FISH of this study.

Genes Names (Gene ID)	Sequences
TGase1 (ncbi_113823934)	AGGGAGATATG+TGCCGTCATGT+TCATT
TGase (ncbi_113823945)	ACGGCGT+TGTAGGCTGGCTGGAAGT
CTL-2 (ncbi_113804553)	GCTCG+TAGTGG+TAGCAGGCCGAAGT
SVC5 (ncbi_113809352)	GGCAGCAACCAGGG+TAAACCAAGGAC
CrustinL (ncbi_113816267)	GCACCTACCGGGCT+TGCAGCAG
Crustin (ncbi_113801825)	GTAGTCGT+TGGAACAGGT+TGTGGGG
Notch1 (ncbi_113814237)	GGGCAACACCT+TCCCGTCACCGA
Ast2 (ncbi_113822052)	ATGTA+TCCGTAAGGACAA+TCTGAAGGCC
CHF (ncbi_113802754)	CAATA+TCCGCAGTAATCAACA+TCAACCC
Negative control	TGCTTTGCACGGTAACGCCTGTTTT

### Cell sorting of hemocytes

To investigate the expression characteristics of critical genes in hemocyte morphological subpopulations, we first analyzed and sorted total hemocytes using FACSAria Fusion flow cytometry (BD, USA). The hemocytes of 10 shrimp were drawn and analyzed by flow cytometry. The side-scatter parameters and forward-scatter parameters (SSC and FSC) were used to distinguish different morphological subpopulations of hemocytes based on their size and granularity. In the FSC/SSC two-dimensional space, two main populations were detected, the Region1 (R1)-small/granular simple population defined as agranulocytes and the Region2 (R2)-large/granular complex population defined as granulocytes. After sorting the total hemocytes according to R1 and R2 regions, the different subpopulations were stained with May-Grunwald and Giemsa staining solutions (Solarbio) in order to observe the composition of the cells.

### QRT-PCR and FISH of selected genes from sorted hemocyte subpopulations

The total RNA of sorted hemocyte subpopulations were separately extracted using TRIzol Reagent^®^ reagent (Invitrogen, USA), and the quality and quantity were detected by a Nanodrop 8000 spectrophotometer (Thermo Scientific, MA, USA). The single-strand cDNA was synthesized by M-MLV reverse transcriptase reagent Kit (Promega). Then the expression profiles of related genes in hemocyte subpopulations were detected by qRT-PCR with specific primers (shown in **Table 2**). The 18S rRNA was used as the reference gene. The qRT-PCR was performed in triplicate using ChamQ Universal SYBR qPCR (Vazyme) in a LightCycler^®^480 II Real Time PCR System (Roche, Basel, Switzerland), following the manufacturer’s protocol. The relative gene expression levels were calculated by the ΔΔCT method against 18S rRNA. For FISH, the obtained hemocyte were fixed and settled onto slides according to the previous procedure, and nucleic acid probes of selected genes were incubated. Then the slides were observed by fluorescence microscopy.

**Table 2:** Primers used in qPCR of this study, related to **Figure 6**.

Genes Names (Gene ID)	Primers	Sequences (5’-3’)
TGase1 (ncbi_113823934)	F	GTGACCCGAAGGAACCAGCC G
	R	AACTGCAGCGAGAACATACTCCTC
TGase (ncbi_113823945)	F	CGAGATGTACGAGCTGGTCGAG
	R	GGACGAGGACCGAAGGAGAAG
CTL-2 (ncbi_113804553)	F	CCACTACTTCTACATTAGCGACGAGG
	R	TCGAAAAGCATCAGGTGCTCAC
SVC5 (ncbi_113809352)	F	GCGCCGACAAGAAGGCAG
	R	AGGCCGAACGGAGAACAGG
CrustinL (ncbi_113816267)	F	CGGTGGCGTCTTCCCAG
	R	TGCAGTAATTGCAGTTGAATCCGC
Crustin (ncbi_113801825)	F	TCTTGGAACTGGCACAAGCGAC
	R	AACGTGGGCATGTGGGAC
Notch1 (ncbi_113814237)	F	GTTGCCCAAGATGCTCCTCC
	R	CCTTGGCCTTGTGCGTTG
Ast2 (ncbi_113822052)	F	CCTCCAGTCAGAGGTTTTGTATTGCA
	R	AACTTCAGCGAACTTCCTCACAG
CHF (ncbi_113802754)	F	GTTGGGTCTATCTGAGGTGGACAATG
	R	CCTTTCCACTTTTCTCAATAGCTGGAG
18S rRNA (AY438005)	F	ACAATGGCTATCACGGGTAACG
	R	CTGCTGCCTTCCTTAGATGTGGTA

F, Forward primer; R, Reverse primer.

### Statistical analysis

Statistical analysis was performed using GraphPad Prism. The data was expressed as the mean ± SD, n=3. Unpaired Student’s t-test was used to determine statistically significant differences, and *p* < 0.05 was considered significant. Date analysis and graph plotting were conducted with the OmicShare online tool (https://www.omicshare.com/).

## Results

### scRNA-seq of hemocytes from healthy *L. vannamei*

**Figure 1:**
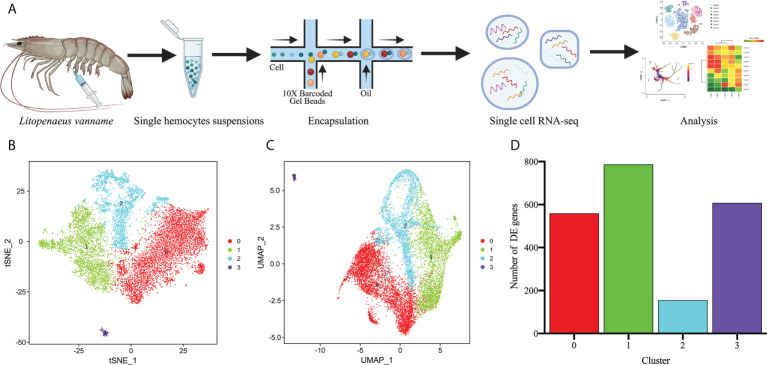
Single-cell RNA-Seq of *Litopenaeus vannamei* hemocytes reveals diverse cell types. **(A)** Overall workflow for schematics of the microfluidics-based scRNA-seq. **(B)** The t-Distributed Stochastic Neighbor Embedding (t-SNE) dimensional reduction was used to visualize the classification results of *L. vannamei* hemocytes. The hemocytes were clustered into four populations using the clustering algorithm based on graph theory. **(C)** The uniform manifold approximation and projection (UMAP) was utilized for the re-clustering analysis and visualization of hemocytes. See **Supplementary Figure 2**. **(D)** Statistics on the number of differentially expressed genes among different hemocyte clusters.

The shrimps used for hemocytes preparation were free of specific pathogenic infections by microscopic observation and bacteriological examination. To perform scRNA-seq of *L. vannamei* hemocytes, the hemocytes of three shrimp were harvested and mixed as one sample, and then the library construction and sequencing analysis were conducted using 10x Genomics platform and Illumina NovaSeq sequencer (**Figure 1A**). By filtering out ineffective cells and low-quality genes, we finally obtained 9896 valid hemocytes for subsequent analysis, each with a median unique molecular identifier (UMI) of 5733.5 and a median gene counts of 1199 (**Supplementary Figure 1**; **Supplementary File 1**). The filtered datasets were visualized and analyzed using principal component linear dimensional reduction and the *t*-distributed stochastic neighborhood embedding (t-SNE) tool (44, 45). The unsupervised cluster detection algorithm (SEURAT) grouped hemocytes into four cell clusters based on the similar gene expression profiles (46) (**Figures 1B, C**). Details of the four cell clusters obtained were as follows: Cluster 0, 4706 cells (47.55%), with a median gene value of 952 and a median UMI of 4066.5 per cell; Cluster 1, 2818 cells (28.48%), with a median gene value of 1552.5 and a median UMI of 8675 per cell, Cluster 2, 2253 cells (22.77%) with a median gene value of 1237 per cell and a median UMI of 10988, and Cluster 3, 119 cells with a median gene value of 1107 per cell and a median UMI of 3251 (**Figure 1D**; **Supplementary Figure 2**).

### Identification of hemocyte clusters

**Figure 2:**
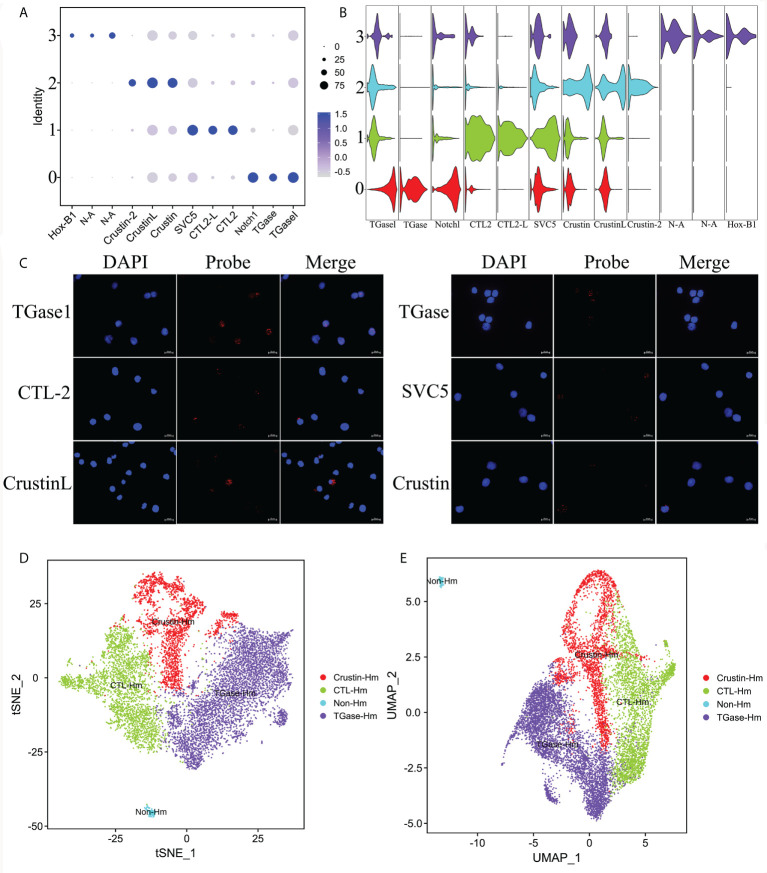
Identification of highly specific cell clusters marker genes and definition of cell clusters. **(A)** Dot plots showing the expression of three specific cell type marker genes across all clusters. Color gradient of the dot represents the expression level, while the size represents percentage of cells expressing any gene per cluster. **(B)** Stacked violin plots showing the expression of 10 commonly used cell type marker genes across all clusters. See **Supplementary Figure 3**, **Supplementary File 3**. **(C)** Validation of the specific marker genes expression in hemocytes by FISH. **(D)** The t-SNE dimensional reduction was used to visualize the classification results of hemocytes. **(E)** The UMAP was utilized for the re-clustering analysis and visualization of hemocytes.

To identify the four types of hemocyte clusters obtained, we first used the Seurat ‘FindMarkers’ tool to predict the TOP20 genes for each cluster (**Supplementary Figure 3**), which were further screened to define highly specific marker genes for each cell cluster, and ultimately a number of potential marker genes were identified for the four hemocyte clusters (**Supplementary Figure 4**). The specific marker genes for Cluster 0 included hemocyte transglutaminase (TGase1), hemocyte transglutaminase (TGase) and neurogenic locus notch homolog protein 1 (Notch1) (**Figures 2A, B**). TGases are the key enzymes in organisms that play critical roles in coagulation and other biochemical processes involving post-translational protein remodeling (47, 48). In crustaceans, TGases act as hemocyte coagulants by cross-linking coagulation proteins, and they also could regulate the expression of immune-related genes such as lysozyme (49, 50). Two types of TGase have been discovered in *L. vannamei* and studies have shown that they were both involved in hemocyte coagulation (49, 51). It was also reported that TGase was one of the immature hemocyte markers in crayfish, and once the TGase around immature hemocytes is digested, hemocytes begin to migrate outward and differentiate into mature hemocytes (52, 53). Notch1, one of the major functional proteins in the notch signaling pathway, is widely associated with cell proliferation, differentiation, activation and innate immunity (54–56). Notch1 mediates the differentiation of *Drosophila* crystal cell clusters and also participates in the coagulation function performed by crystal cells (57–59). In shrimp, it is hypothesized that Notch1 might mediate the differentiation of Cluster 0 cells and also perform a certain function in hemocyte coagulation. The high expression profiles of TGase and Notch1 implied that the Cluster 0 is likely to be the main cellular type for hemocyte clotting and is in the early stage of hemocyte differentiation. C-type lectin 2 (CTL-2), C-type lectin 2 like (CTL-2L), single VWC domain protein 5 (SVC5) were identified as the marker genes for Cluster 1(**Figures 2A, B**). Lectins are considered the primary candidates for pattern recognition receptors (PRRs) in innate immunity because of their ability to bind specific carbohydrates on the surface of microorganisms (60–62). Calcium-dependent lectins (C-type lectin) are members of the typical lectin family with two or three pairs of disulfide-bonded characteristic carbohydrate recognition structural domains (CRDs) that exert pathogen recognition and clearance (62). A variety of C-type lectins with broad antibacterial and antiviral activity have been characterized in invertebrates, such as *Penaeus japonicus* and *L. vannamei* (63–65). SCV5 is a member of the von Willebrand factor type C (VWC) domains protein family, which play an important role in response to pathogen infestation (66, 67). Based on the analysis of key genes described above, we speculated that Cluster 1 might be the primary cell type that could recognize pathogens. Crustins (Crustin like, Crustin, Crusin 2) are specific marker genes of Cluster 2 (**Figures 2A, B**). As a family of antimicrobial peptides (AMP) rich in cysteines and with several structural domains of whey acidic proteins (WAP), Crustin is widespread in decapod crustaceans (68–70). Due to its important antimicrobial role, Crustin is also known as a key component of the innate immune system of shrimp (71, 72). Thus, Cluster 2 might be the hemocyte type that perform the primary immune functions. Cluster 3 is the most specific kind of hemocytes, with the lowest number of cells. In the downscaling analysis, Cluster 3 is the most distant from the other cell clusters and its genes are largely unannotated, most likely being non-hemocyte (**Figures 2A, B**). The non-hemocyte has also been reported in previous single cell sequencing study of *Drosophila* hemocytes (34). The results of RNA-FISH further confirmed the distribution of the above-mentioned marker genes in hemocytes (**Figure 2C**). In summary, we defined the above four hemocyte clusters as TGase^+^ cells, CTL^+^ cells, Crustin^+^ cells and non-hemocyte, respectively (**Figures 2D, E**).

### Functional prediction of hemocyte clusters

To further explore the functional characteristics of the above hemocyte clusters, we first performed GO functional annotation and KEGG pathway enrichment analysis of the differential genes among the clusters. According to the GO functional annotation, the differential genes of the TGase^+^ cell cluster were mainly assigned to 13 categories of biological processes, six categories of molecular functions and 21 categories of cellular components. The KEGG pathway enrichment showed that the differential genes of the TGase^+^ cell cluster were significantly enriched in 21 biological pathways such as ribosome (ko03001), spliceosome (ko03040) and VEGF signaling pathway (ko04370). The differential genes of the CTL^+^ cell cluster were mainly enriched in GO annotations to 14 categories of biological processes, 20 categories of molecular function and 14 categories of cellular component and were assigned to 18 biological pathways including oxidative phosphorylation (ko03050) in the KEGG functional enrichment The differential genes of the Crustin^+^ cell cluster were enriched to a total of 15 classes of biological processes and nine classes of molecular function, and the cellular components were not enriched. The KEGG enrichment result of the Crustin^+^ cell cluster showed that its differential genes were mainly enriched in seven pathways including antigen treatment (ko0462) (**Supplementary Figure 5**; **Supplementary File 2**). The GO term and KEGG pathways enriched for non-hemocyte differential genes was too complex, again illustrating the specificity of this cluster. As previously reported, GO and KEGG functional annotation did not provide specific functions of the cell clusters, probably due to the lack of information on the annotation of the relevant genes (73).

**Figure 3:**
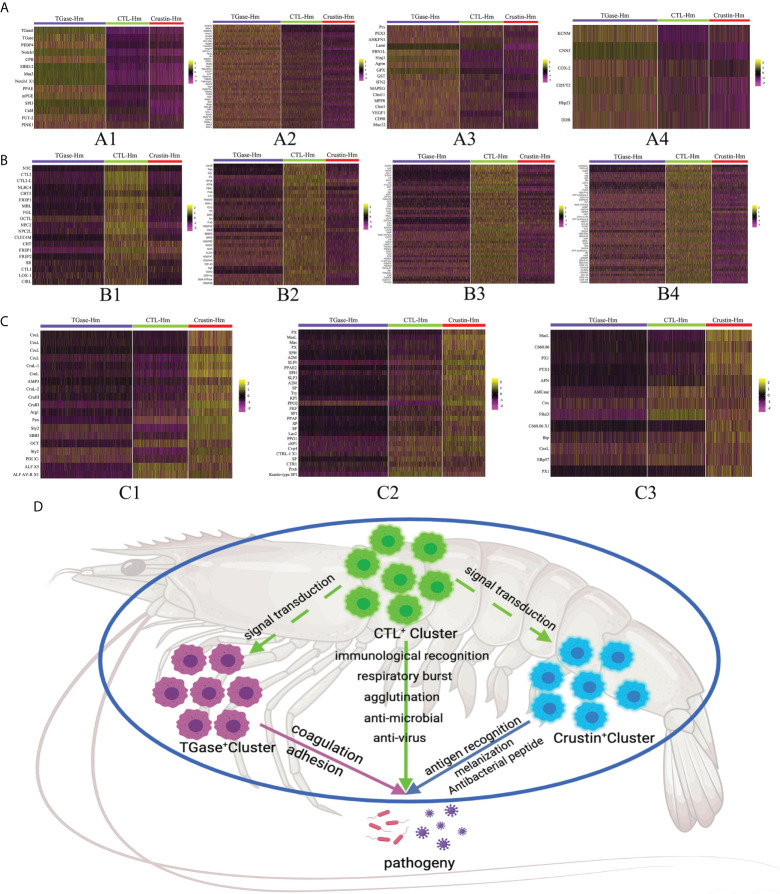
ScRNA-seq unveils the functional characteristics of different hemocyte clusters. **(A–C)** The heatmaps shows the expression profiles of the different functional feature genes in Cluster 0, Cluster 1 and Cluster 2 (TGase^+^ cluster, CTL^+^ cluster and Crustin^+^ cluster), respectively. Color gradient represents the expression level of each single cell (**Supplementary File 5**). **(D)** The schematic exhibits the functional characteristics and interactions of the three hemocyte clusters.

Then, we performed more detailed analysis of the differential genes that were significantly upregulated in each cluster to comprehensively reveal the functional characteristics of the four types of cell clusters (**Supplementary File 3**). Based on the scRNA-seq analysis, we found that TGase^+^ cells significantly overexpressed genes associated with coagulation, such as phosphatidylethanolamine-binding protein 4-like (PEBP4), carboxypeptidase B-like (CBP) and single insulin-like growth factor-binding domain protein-2 (SIBD-2) (**Figure 3A**). These molecules could act on TGase to promote its coagulation function or participate in hemocyte coagulation by regulating the cross-linking of other coagulation proteins (74–77). TGase has been reported to be secreted by the exosome pathway to exocytosis for its function. Significantly high expression of genes involved in the exosome pathway was also found in TGase+ cells, suggesting that this cluster is prominently functioned by the TGase (**Figure 3A**) (78–81). Meanwhile, TGase^+^ cells also showed high expression of genes related to cell adhesion, which is capable of aiding hemocyte coagulation and the wound healing process (**Figure 3A**) (82–84). Numbers of genes involved in blood coagulation of higher animals were also highly expressed in the cluster, but the roles of their auxiliary coagulation function remain to be investigated (**Figure 3A**) (85, 86). Based on the results of these analyses, we ventured to speculate that TGase^+^ hemocyte primarily perform the clotting function.

The analysis of highly expressed genes showed significantly upregulated expressions of lectin-like genes in CTL^+^ cells, including C-type lectin, fibrinogen and serine protease-like proteins (**Figure 3B**). Lectins are the most thoroughly studied pattern recognition receptors in shrimp, sensing pathogen invasion and activating the immune response. Lectins are also involved in other biological processes, such as intercellular interactions, protein synthesis and signal transduction (87, 88). Moreover, oxidative stress genes were found to be highly expressed in CTL^+^ cells along with certain immune-related genes (**Figure 3B**). Oxidative stress is the process of excess reactive oxygen species (ROS) production resulting from the imbalance between cellular oxidative and antioxidant reactions. ROS is produced as an intermediate product of oxidative respiration in mitochondria as well as many metabolic processes, which is involved in the defense of immune cells against invading pathogens (89, 90). The presence of significantly high expression of immune-related genes, such as lysosomal enzyme-related molecules and anti-lipopolysaccharide factors (ALFs), indicated that CTL^+^ cell cluster play the anti-infective function and generate immune responses. Further analysis revealed that the expression of cellular metabolism-related molecules, especially oxidative phosphorylation-related genes, were significantly higher in the CTL^+^ cell than in other clusters (**Figure 3B**). Oxidative phosphorylation is the key component of mitochondrial respiration and is intimately linked to ROS production, again suggesting that this cluster is active in oxidative stress processes (91). Combined with the significantly upregulated genes described above, the CTL^+^ cell cluster is likely to be the key cell type that recognizes pathogens and produces ROS and immune molecules for anti-infective functions, while the high expression of its signal transduction-related genes allows the cluster cells to transmit triggering signals to stimulate other cell types to perform different immune functions (**Figure 3B**).

The Crustin^+^ cell cluster had the lowest number of significantly up-regulated genes compared to the two previous hemocyte clusters. Analysis revealed that the genes significantly overexpressed in this cluster consisted mainly of various antimicrobial peptide (AMPs), and molecules associated with the phenoloxidase system (**Figure 3C**). AMPs, as the main molecules of the innate immune response in crustaceans, are able to fight against various pathogenic invasions (92). The phenoloxidase system is essential for the production of melanin in shrimp hemocytes, preventing further infection by pathogens (93). The high expression of these genes implied that the Crustin^+^ cell cluster is the main cell type exerting immune responses. Also, we found that some of the classical pattern recognition receptors, such as Toll-like receptors and F-type lectins, were highly expressed in Crustin^+^ cells, which indicated that this cell cluster also functions in immune recognition (**Figure 3C**). The high expression of immune recognition genes in this cluster might be required for AMP molecules production and activation of the phenoloxidase system (94, 95). Based on the functional characteristics of these highly expressed genes, we considered that the Crustin^+^ cell cluster is the main immune response cell and plays the critical anti-disease function.

In summary, we hypothesized that TGase^+^ cells are the main cell type in hemolymph that function as hemocyte coagulation and cell adhesion. CTL^+^ cells are the main immune recognition cells that could recognize foreign pathogens and transmit immune signals to other cell clusters, as well as acting in certain immune response roles. And Crustin^+^ cells are the main immune response cells that play essential roles in fighting pathogens (**Figure 3D**). Taken together, the three types of cells form the shrimp hemocyte immune system that defends against pathogenic invasion.

### Pseudotemporal ordering and trajectory reconstitution of hemocyte lineages

**Figure 4:**
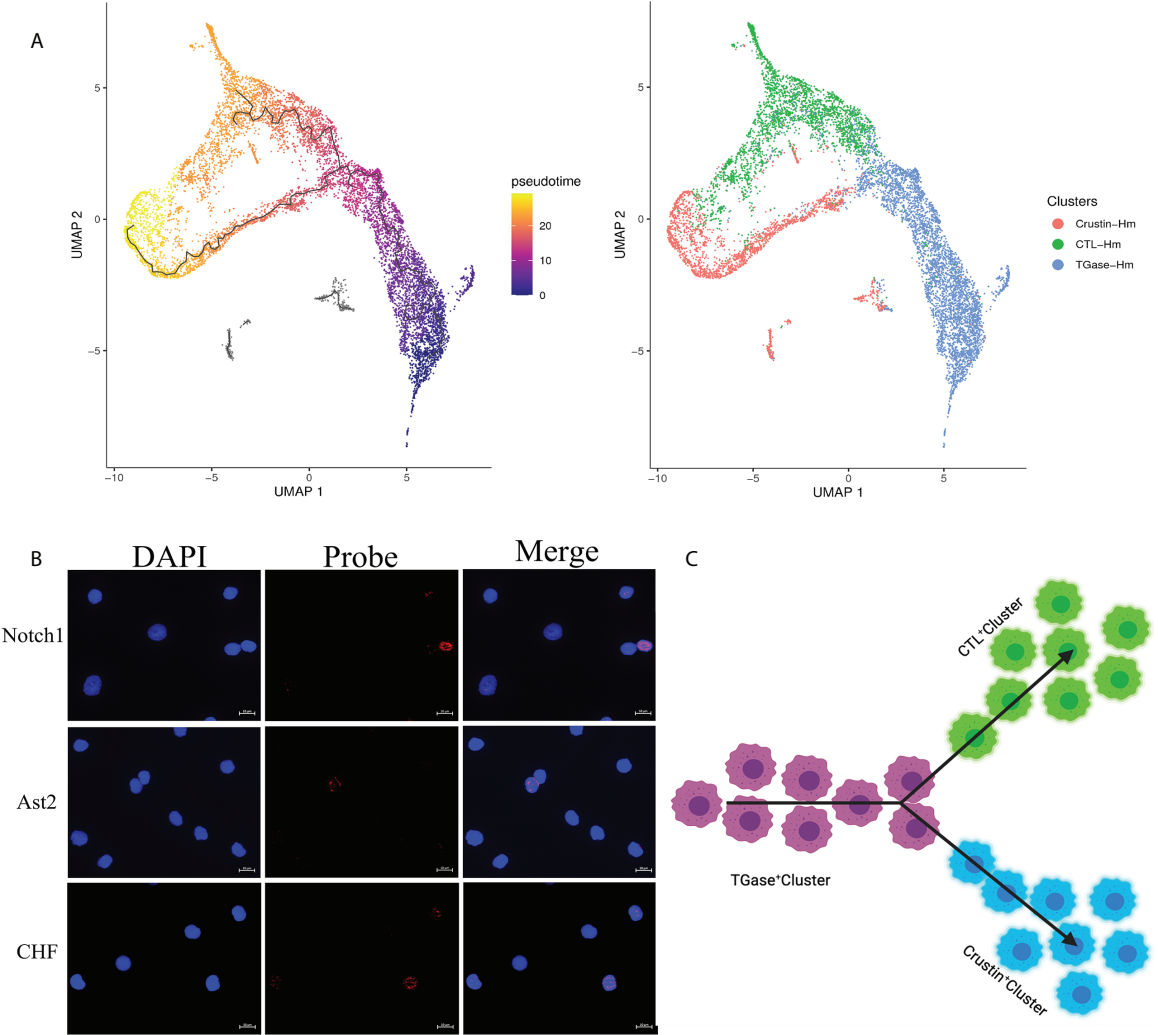
Pseudotemporal ordering of cells delineates hemocytes lineages. **(A)** Monocle 3 was used to track hemocytes over pseudotime on the 10X data sets. Visualization of cell clusters (from Figure 1C) onto the pseudotime map. **(B)** Validation of the key differentiation genes (Notch1, Ast2 and CHF) expression in hemocytes. **(C)** Schematic showing potential lineage flow from the TGase^+^ cluster to CTL^+^ cluster and Crustin^+^ cluster.

To investigate the kinetics of hemocyte differentiation and to reveal the cell lineage relationships among different hemocyte clusters, we used Monocle3 functions to perform pseudotime analysis and cell lineage-tree reconstruction of the obtained hemocyte clusters. The pseudotime analysis assigned TGase^+^ cluster as the starting point of the pseudotime intervals and showed that two lineages (CTL^+^ cluster and Crustin^+^ cluster) emerge from the start point (**Figure 4A**). Meanwhile, the pseudotime analysis showed distinct intermediate states of cells among the three types of hemocyte clusters, suggesting that the differentiation process of hemocytes was continuous, which was consistent with the hematopoietic mechanism of crustacean (53). We analyzed the cell differentiation and cell cycle related highly expressed genes of each cell cluster and the results further confirmed the accuracy of the pseudotime analysis. It has been previously clarified that high expression level of TGase in TGase+ cells could be served as a marker for immature cells in crayfish (52, 96), suggesting that it would be logical to consider TGase^+^ cluster as a starting point for pseudotemporal analysis. The Notch signaling pathway functions as a crucial pathway for *Drosophila* hemocytes differentiation (97), and the high expression of differentiation-related genes in this cluster suggest that TGase^+^ cells are likely to act as pre-differentiated cells with differentiating and maturing in response to the differentiation-related signaling pathways. However, there is no clear evidence of whether the Notch signaling pathway contributes to cell development in crustaceans, particularly in shrimp (98). We also found a number of cell cycle-related genes exhibited highly expressions in TGase+ cells, such as AKB and GATA6 (**Supplementary Figure 6**; **Supplementary File 4**). These genes are extremely important in regulating the cell cycle by stabilizing chromosomes, promoting DNA replication and controlling the process of chromosome and cytoplasm splitting during cell division (99–101). These results indicated that the TGase^+^ cell cluster has the ability to divide continuously, which is in line with the inference that the cluster is in the early stage of differentiation and confirms the plausibility of this cluster as the presumed starting point for cell differentiation. Based on the pseudotemporal differentiation analysis, the CTL^+^ cluster and Crustin^+^ cluster were at the end of the differentiation spectrum as terminally differentiated hemocytes. In the differential genes analysis, a lower number of differentiation-related genes was found in these two types of clusters. However, pivotal genes associated with maturation of crustacean hemocytes were detected both in two cell clusters (**Supplementary Figure 6**; **Supplementary File 4**). Astakine2 (Ast2), a homolog of vertebrate hematopoietic factors, is highly expressed in the CTL^+^ cluster and plays a key role in the maturation of shrimp hemocytes, while Ast2 has been shown to be essential for the differentiation of the granulosa cell lineage in crayfish (102). VEGF-3 and Sulf, which have been shown to be involved in *Drosophila* hematopoietic cell differentiation and maturation (103, 104), are also highly expressed in CTL^+^ cluster. In addition, several molecules implicated in precursor cell differentiation and maturation in vertebrates such as BMPER, Elk-3 and HDAC were also significantly upregulated in the CTL^+^ cluster (105, 106). Crustacean hematopoietic factor (CHF) and its analogue were highly expressed in the Crustin^+^ cluster, which are critical for the maturation of hematopoietic tissue cells and is an important regulatory molecule in the development of the crayfish granulocyte lineage (**Supplementary Figure 6**; **Supplementary File 4**) (107). The presence of these maturation-related molecules implied the existence of cells in the intermediate state of differentiation and suggested that CTL^+^ cluster and Crustin^+^ clusters are indeed the end-state cells of the shrimp hemocytes lineage development. Collectively, the results of the pseudotime analysis identified TGase^+^ cluster arrested at an early developmental state with the ability to differentiate into CTL^+^ clusters and Crustin^+^ clusters, and that this differentiation process was continuous (**Figures 4B, C**).

### Diversity of hemocyte sub-clusters and their transcriptional dynamics

The previous pseudotime analysis found continuous differentiation among the obtained three hemocyte clusters, along with intermediate state differentiated hemocytes types. In order to further obtain the cell types of intermediate state, we subclassified TGase^+^ cluster, CTL^+^ cluster and Crustin^+^ cluster, acquired 10 classes of sub-clusters and resolved their gene expression profiles (**Supplementary File 5**) and differentiation relationships.

### TGase^+^ cluster

**Figure 5:**
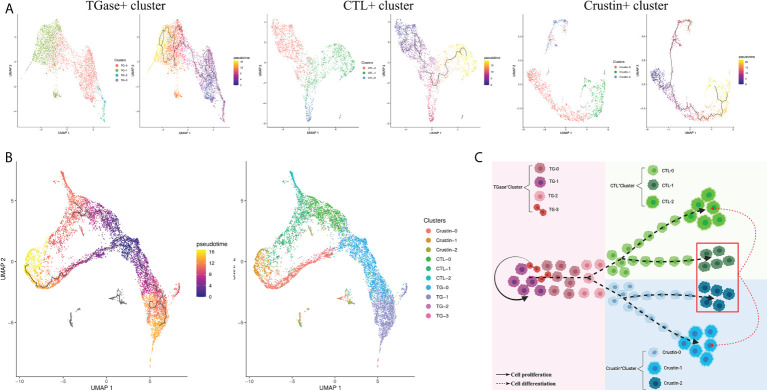
Diversity of hemocyte populations and their potential lineage. **(A)** Single-cell trajectory for the sub-clusters of TGase^+^ cluster, CTL^+^ cluster and Crustin^+^ cluster. **(B)** Visualization of all sub-clusters onto the pseudotime map. **(C)** Schematic exhibiting potential lineage flow from the oligopotent state of TGase^+^ cluster to mature cell types (CTL^+^ cluster and Crustin^+^ cluster) with their intermediates.

Sub-clustering of the TGase^+^ cluster obtained a total of four subclusters, named TG-0, TG-1, TG-2 and TG-3 (**Supplementary Figure 7**). The differential genes analysis of the four subclusters revealed that TG-0 was highly expressed the genes involved in cell cycle and differentiation, such as ATRX, TTF1 and LIG1 (**Supplementary Figure 8**) (108–110). In parallel, TG-0 was assigned as the presumed starting point for differentiation in a pseudotime differentiation analysis of the TGase^+^ cell subcluster, suggesting that TG-0 might be the differentiated precursor cells for the TGase+ cluster with the ability to continue differentiation (**Figure 5A**). Compared to other subclusters, TG-1 has high in expressions of hemocyte coagulation-related molecules, especially TGase, which indicated that this subcluster might be the main sub-type that exerts coagulation function. Some genes that are involved in the regulation of cell differentiation and division also appeared to be hyper-expressed, and TG-1 should be able to regulate the differentiation or division state of certain cells. TG-2 showed a different differentiation direction from TG-1 in the reconstruction of differentiation trajectories and was enriched for genes that promote cell migration to the correct location during cell differentiation (**Supplementary Figure 8**). In addition, TG-2 showed upregulated expression of several functional genes of CTL^+^ cluster and Crustin^+^ clusters, implying that it is likely to be an intermediate state cell in the differentiation stage from TGase^+^ clusters to CTL^+^ or Crustin^+^ clusters (**Supplementary Figure 8**). We determined TG-3 as the cell cycling or self-renewing state of TGase^+^ cluster based on the expression of genes which are indispensable marker of the cell cycle such as CCNA1, zmcm3 and INCENP (111–113).

### CTL^+^ cluster

The CTL^+^ clusters were re-clustered to obtain CTL-0, CTL-1 and CTL-2 subclusters, and the three subclusters were analyzed for pseudotime differentiation (**Figure 5A**; **Supplementary Figure 7**). The results show that CTL-0 was assigned as the starting point for pseudo-temporal differentiation. And molecules acting on invertebrate hemocyte differentiation such as Notch1 and VEGF3 were highly expressed in CTL-0 (97, 103), which confirmed the plausibility of the pseudotime differentiation for the CTL^+^ cluster (**Supplementary Figure 8**). This subcluster also showed high expression levels of genes related to mitochondrial function. In the CTL-1 subcluster, we found high expressions of immune defense genes, especially AMPs and molecules of regulating the phenoloxidase system (**Supplementary Figure 8**). We speculated that this subcluster is probably analogous to Crustin^+^ cluster with immune function. The high expression of CHF in CTL-1 confirmed our speculation that the subcluster is likely to be an intermediate state cell type which differentiates into distinct cells in response to various differentiation stimulating molecules (**Supplementary Figure 8**) (107). The CTL-2 showed relatively simple gene expression profiles with signature genes largely engaged in immune recognition as well as signal transduction, which supported the view that the subcluster is the main functional cells of CTL^+^ cluster (**Supplementary Figure 8**).

### Crustin^+^ cluster

The Crustin^+^ cluster was divided into three subclusters, Crustin-0, Crustin-1 and Crustin-2 (**Supplementary Figure 7**). The cell differentiation-related molecules, particularly CHF, were significantly highly expressed in Crustin-0, suggesting that the subcluster might be used as putative starting point for differentiation of Crustin^+^ cluste (**Figure 5A**). Crustin-1 was probably the effector cell type of the Crustin^+^ cluster, and its high expression profiles of AMPs and phenoloxidase-related molecules lent support to this conclusion (**Supplementary Figure 8**). Interestingly for Crustin-2, the subcluster had both cellular differentiation genes such as Notch1, VEGF3 and functional genes in other clusters like TGase and CTL. Crustin-2 seemed to be analogous with CTL-1, and the subcluster had divergent differentiation directions in response to stimulation by differentiation factors (**Supplementary Figure 8**).

Based on the above analysis, we performed a pseudo-temporal differentiation analysis of the 10 secondary subpopulations here, and the results were presumably in line with what we had hypothesized (**Figure 5B**). TG-0, as the putative starting point for differentiation, could differentiate into TG-1 for hemocyte coagulation on the one hand, and TG-2 for migration and differentiation into other cell clusters on the other. TG-3 acts as self-renewing cells for TGase+ cluster and is capable of dividing and proliferating. TG-2 is progressively differentiated into CTL-0 in response to stimulation by Notch-related molecules, and CTL-0 further differentiate into mature CTL-2 for immune recognition and signal transduction. Crustin-0 is also supposed to differentiate and migrate from TG-2, which differentiates into mature Crustin-1 under the involvement of CHF for performing immune defense functions. Crustin-2 and CTL-1 are similar in that both might differentiate into different subclusters in response to stimulation by differentiation factors (**Figure 5C**).

### The relationship between hemocyte clusters and morphology

**Figure 6:**
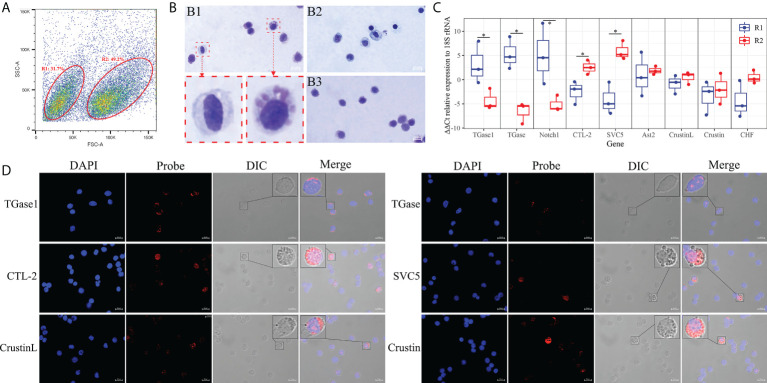
Morphological analysis of hemocytes clusters. **(A)** FACS analysis of hemocytes. Based on the FSC and SSC, two regions (R1 and R2) were sorted, respectively. **(B)** Hemocytes and cell subpopulations were stained by May-Grunwald and Giemsa dye. B1: hemocytes. B2: R1 sorted hemocytes. B3: R2 sorted hemocytes. **(C)** Differential expression analysis of highly specific cell clusters gene between R1 and R2 of hemocytes sorted using FACS. **(D)** Validation of the highly specific cell clusters gene among hemocyte subpopulations by FISH. The asterisks on the bars represent the statistical difference of the hemocyte subpopulations.

To correlate the obtained hemocyte clusters with morphological subpopulations, we sorted the hemocytes using flow cytometry. Two types of hemocyte subpopulations were defined and sorted according to relative cell size (forward scatter plot, FSC) and subcellular organelle characteristics/relative cell granularity (lateral scatter plot, SSC) (**Figure 6A**). The FCM showed that the hemocytes in region 1 are small in relative cell size and have the low intracellular complexity (R1, 31.7% ± 5.3%). Region 2 (R2) contained larger cells with high intracellular complexity (49.2% ± 4.6%). The two types of subpopulations obtained by sorting were stained to visualize histological features. The hemocytes in R1 were relatively small, with a large nucleoplasmic ratio, and the interior of the cell was basically free of granular material, similar to the morphological characteristics of hyalinocytes (**Figure 6B**) (73, 114). The hemocytes in R2 were slightly larger, with a smaller nucleoplasmic ratio and more granules inside the cells, resembling the morphological characteristics of granule-containing hemocytes (**Figure 6B**) (73, 114).

In order to determine the relationship between the two hemocyte subpopulations obtained by flow cytometry sorting and the hemocyte clusters gained by scRNA-seq, qRT-PCR was performed to explore the expression characteristics of marker genes and crucially differentiated genes. The results showed that marker genes of TGase^+^ cell cluster such as TGase1 and Notch1 were mainly expressed in hyalinocytes (R1), while CTL^+^ as well as Crustin+ cell clusters marker genes like CTL-2, SVC5, and Crustin were primarily expressed in granule-containing hemocytes (R2) (**Figure 6C**; **Supplementary File 6**). Moreover, the distribution of important genes in different subpopulations were further determined by RNA-FISH. TGase1 and TGase were predominantly found in hyalinocytes. CTL-2, SVC5, CrustinL and Crustin were mainly observed in granule-containing hemocytes (**Figure 6D**). Based on these data, we can reasonably conclude that the TGase^+^ cells are morphologically characterized as hyalinocytes and the CTL^+^ cells and Crustin^+^ cells are likely to be the granule-containing hemocytes.

## Discussion

The hemocytes are the vital immune cells in shrimp, thus the classification and identification of hemocytes are essential for better elucidating the innate immune mechanisms of shrimp. Studies on the classification of shrimp hemocytes began as early as the 19th century (14, 115). Although the currently agreed classification system for hemocytes is based primarily on morphological characteristics, the different criteria researchers have adopted to descript and identify hemocyte has led to a highly confusing classification system for hemocytes (11, 116–118). Subsequent researchers have proposed interpretation of hemocyte types through multiple perspectives on the basis of cell morphology combined with cytochemistry. Some studies have also established new means for hemocyte classification according to biochemical markers on the cell surface, with reference to the classification criteria adopted for mammalian immune cells (19, 20). Yet, there is still no consistent agreement on the classification of crustacean hemocytes. In addition, there is much controversy about the differences in immunological functions performed by different hemocyte subpopulations, such as cytotoxicity (11, 117, 119). The developmental characteristics of circulating hemocyte lineages have not been fully elucidated (21, 22). In the present work, the gene expression characteristics of single hemocyte were obtained by high-throughput sequencing with the aid of scRNA-seq technology, and the classification features of hemocytes from healthy *Litopenaeus vannamei* was comprehensively analyzed in terms of molecular features, with a view to elucidating the functions of different hemocyte clusters and their differentiation relationships.

Through scRNA-seq analysis, four hemocyte clusters were identified based on their differential gene expression profiles. Due to the extremely small cell number of Cluster 3 and the fact that most of the highly expressed genes in the cluster have not been annotated, we have tentatively defined it as a non-hematopoietic cluster. Similar non-hemocytes cluster have been also found in *Drosophila* blood and might be impurity cells (34). We made functional definitions of the other three hemocyte clusters using specific marker genes and functional genes, identified the cell types and found that they do exhibit heterogeneous immune functions (120–123). Different numbers of cellular clusters have also been identified in previous studies on the scRNA-seq of shrimp hemocytes. Six cell clusters were identified in healthy *Marsupenaeus japonicus* that had not been subjected to any treatment (73). Under ammonia stress, hemocytes of *Penaeus monodon* were divided into seven main cell groups (124). Following stimulation by hemocyte activating factors, hemocytes of *L. vannamei* were classified into prohemocytes, granulocytes, monocytic hemocytes, transitional cells and germ-like cells (125). The difference in hemocyte classification results of scRNA-seq might be due to species specificity. In addition, our results did not find cell cluster with significantly higher expression of antiviral molecules such as Vago5 in healthy *L. vannamei* hemocytes, nor were the cluster reported in *P. monodon*, but it appeared in the second subpopulation of healthy *M. japonicus* hemocytes. Differences in hemocyte clustering might also be the result of various treatments, which could lead to significant upregulation of key genes in a cluster, resulting in different classifying outcomes, which is also common in scRNA-seq studies of other species, as reported in the case of hemocytes in *Drosophila* and mosquito (33, 34, 126, 127). It is also possible that the cell clustering differences are attributable to the fact that shrimp hemocytes are themselves a dynamic and changing tissue that are not fully mature, and that shrimp growth status, sampling time, sampling methods and analytical parameters might all contribute to the differences. Nevertheless, all scRNA-seq studies of shrimp hemocytes have emphasized the existence of cell clusters that perform coagulation cascades, kill pathogens, and other distinct immune functions. Given our analysis, it is assumed that CTL^+^ cells could recognize invading pathogens and initiate partial immune functions, while transmitting immune signals to other clusters. Crustin^+^ cells recognize pathogens and receive immune signals, initiating immune response pathways, releasing molecules such as AMP and activating phenoloxidase system to directly kill pathogens. During this process, TGase^+^ cells are stimulated by the immune signals and begin to release cellular agglutination factors that drive coagulation. The three types of hemocytes complement each other and form the shrimp hemocytes immune defense system. Interestingly, our results did not reveal differences in the expression profiles of phagocytosis-related genes among hemocyte clusters, indicating that all types of hemocytes have phagocytic potential (120, 128). In healthy *M. japonicus* hemocytes, the cell type that mainly exerts phagocytosis was also not clearly identified (73). Nevertheless, the hemocytes from treated *L. vannamei* and *P. monodon* exhibited cell clusters that exerted phagocytosis (124, 125), suggesting that the phagocytic cell types might be differentiated from a particular type of hemocytes, which also indicated that different treatments would lead to the differences of cell clustering.

For the purpose of clarifying the functional characteristics of hemocyte morphological subpopulations, we further developed the connectivity between the hemocyte clusters obtained by scRNA-seq with morphological subpopulations. Two hemocyte subpopulations, hyalinocytes and granule-containing hemocytes, were sorted out by flow cytometry, whereas semi-granulocytes and granulocytes did not appear to be identified by flow cytometry (73). May-Grunwald and Giemsa staining of the sorted granule-containing hemocytes was still able to reveal the presence of two classes of granulocytes with different granularity. With the aim of functionally validating the two morphologically distinct subpopulations, we detected the distribution of key functional genes by FISH and qPCR. The results showed that hyalinocytes shared similar gene expression profile with TGase^+^ cluster, suggesting its main role in hemocyte coagulation and hemolymph adhesion. Granule-containing hemocytes highly expressed the genes involved in immune recognition, ROS burst, AMPs and phenoloxidase, indicating they executed immune response functions as CTL^+^ and Crustin^+^ cells. This conclusion is supported by previous studies that found TGase is mainly expressed in hyalinocytes, and semi-granulocytes take the lead in responding to foreign pathogens and AMPs are synthesized and secreted in granulocytes (36, 52, 129). Moreover, based on previously reported marker genes for different morphological subpopulations, recent scRNA-seq analysis of shrimp hemocyte also inferred that hyalinocytes are the main cell type that exert cellular agglutination and granule-containing hemocytes mainly perform the immune defense functions against pathogens (73, 124, 125). However, we still lack direct evidence to develop the connectivity between CLT^+^ cluster and Crustin^+^ cluster with semi-granulocytes and granulocytes. We hypothesized that when pathogens invade, granule-containing hemocytes of shrimp are able to recognize foreign invaders and perform direct immune functions to kill them by releasing lectins, AMPs and other immune functional molecules and activate the phenoloxidase system. simultaneously, the granule-containing hemocytes could transmit immune signals and stimulate hyalinocytes to secrete cellular coagulation factors, thus inducing hemocytes coagulation, encapsulation and nodulation (**Supplementary Figure 9A**).

Subsequently, we mapped pseudotime differentiation trajectories of the obtained hemocyte clusters and their sub-clusters using the Monocle 3 function. By analyzing highly expressed differentiation-related genes, we identified TGase^+^ cluster as being at an early stage of differentiation, capable of migrating and differentiating into CTL^+^ cluster and Crustin^+^ cluster. FISH and qPCR results further found that the TGase served as a marker for immature hemocytes in crustaceans is highly expressed in hyalinocytes (52), indicating that hyalinocytes are arrested at the early stage of hematopoietic differentiation. In hyalinocytes, a significant enrichment of molecules was found to be associated with the Notch signaling pathway that is the regulatory lynchpin of invertebrate hemocytes lineage development (130, 131), implicating that the Notch signaling pathway probably holds central roles in the induction of shrimp hyalinocytes differentiation. The CTL^+^ and Crustin^+^ clusters at the terminal states of the pseudotime differentiation trajectory showed high expressions of Ast2 and CHF respectively, which are two important regulators of granulocyte lineage maturation in shrimp (102, 132). On the basis of these results, we speculated that TGase^+^ cluster (hyalinocytes) are first released in hematopoietic tissue (96) and begin to differentiate with the action of the Notch signaling pathway, followed by gradual development into mature CTL^+^ cluster and Crustin^+^ cluster (granule-containing hemocytes) under the effects of molecules such as Ast and CHF (**Supplementary Figure 9B**). This seems to be verified in shrimp hematopoietic tissues, where the precursor cells of granule-containing hemocytes exhibit characteristics of hyalinocytes (22, 96). This is also consistent with the studies on hemocytes of *M. japonicus* and *P. monodon*, in which cells highly expressing TGase were classified as early differentiating cell types, while cells highly expressing molecules of AMP and proPO were considered to be mature cell types (73, 124). Yang et al. defined that the prohemocytes of *L. vannamei* hemocytes exhibiting high expression of TGase were classified as early differentiated cell types, while they defined monocytic hemocyte and granulocytes with high expression of molecules such as AMP were classified as late differentiated cell types (125). Taken together, these evidence suggested that there are two developmental lineages of shrimp hemocytes that differentiate and mature from hyalinocytes into different granule-containing hemocytes, and semi-granulocytes and granulocytes still show some differentiation association (96, 102, 107, 125). However, the differentiation relationship between granulocytes and semi-granulocytes still cannot be determined, since we are unable to arbitrarily judge how the CTL^+^ cluster and Crustin^+^ cluster correspond to granulocytes and semi-granulocytes.

To further characterize the presence of oligopotent/initial state cells, intermediate state cells and terminally differentiated effector cells in the hemocytes, we performed sub-clustering analysis of the three hemocyte clusters. The results showed that less than 1% of the TGase^+^ cluster (hyalinocytes) are in the cytokinesis cycle (TG-3). This subcluster was in an oligopotent state and might be critical for the self-renewal of TGase^+^ cluster. Previous studies have also discovered a small number of hemocytes undergoing the cell cycle in crustacean hemolymph (9, 125, 133). In parallel, we identified subclusters of hemocyte clusters in transient intermediate states. TG-0 highly expressed the differentiation related genes and was at the putative starting point for pseudotime differentiation analysis, which could be considered the point of initiation of differentiation. TG-2 showed the expression of functional genes of other hemocyte clusters (CTL^+^ cells and Crustin^+^ cells), suggesting the onset of branching points of differentiation. The CTL-0 and Crustin-0 subclusters contiguous to TG-2 actively express the genes that are involved in cell differentiation and migration, indicating that these hemocytes were gradually maturing at this juncture. Furthermore, three effector cell subclusters (TG-1, CTL-2, Crustin-1) were found to exert their specific immune functions. To be noted, the expression of some cell cycle-related genes could still be observed in TG-1, but not in CTL-2 and Crustin-1. Since terminally differentiated cells might require downregulation of cell cycle gene expression to arrest cell division, we hypothesized that CTL-2 and Crustin-1 are terminally differentiated cells whereas TG-1 is not (134, 135). The CTL-1 and Crustin-2 were the most complicated cell, and we assumed that these two types of subclusters are likely to have bidirectional differentiation potential in response to different differentiation factors (21, 136). According to the above analysis, hyalinocytes (TGase^+^ cluster) are most probably dynamically changing hemocytes that do not have sub-clusters of terminally differentiated cells, which acts as differentiation precursor cells and gradually differentiates into mature granule-containing hemocytes with specific functions (CTL^+^ cluster and Crustin^+^ cluster) (**Supplementary Figure 9C**).

This study revealed the functional subtypes and differentiation characteristics of shrimp hemocytes by scRNA-seq and explored the association information between morphological cell subpopulations and functional clusters for the first time, which provided abundant data for the classification, identification and developmental lineages of shrimp hemocyte subpopulations and established a solid foundation for further investigations on the immune functions of hemocyte subpopulations.

## Data availability statement

The datasets presented in this study can be found in online repositories. The names of the repository/repositories and accession number(s) can be found below: NCBI, accession ID: SRR18899521.

## Ethics statement

The animal study was reviewed and approved by the International Guiding Principles for Biomedical Research Involving Animals documented by Guide for the Use of Experimental Animals and the Committee of the Ethics on Animal Care and Experiments at Ocean University of China.

## Author contributions

CC and XT contributed to the conception and design of this study, performed most of experiments and statistical analysis, drafted and revised the manuscript; JX, XS, and HC participated in the design of the study, helped analyzed experiments and data; XT and WZ designed the study, edited the manuscript, and provided reagents and experiment space. All authors contributed to the article and approved the submitted version.

## Funding

This research was financially supported by the National Key Research and Development Program of China (2018YFD0900504, 2019YFD0900101), Qingdao National Laboratory for Marine Science and Technology (QNLM2016ORP0307) and the Taishan Scholar Program of Shandong Province.

## Acknowledgments

We are grateful to Guangzhou Genedenovo Biotechnology Co., Ltd for assisting in sequencing and/or bioinformatics analysis. The schematics were created with BioRender.com.

## Conflict of interest

The authors declare that the research was conducted in the absence of any commercial or financial relationships that could be construed as a potential conflict of interest.

## Publisher’s note

All claims expressed in this article are solely those of the authors and do not necessarily represent those of their affiliated organizations, or those of the publisher, the editors and the reviewers. Any product that may be evaluated in this article, or claim that may be made by its manufacturer, is not guaranteed or endorsed by the publisher.

## Supplementary material

Supplementary Figure 1 Sequencing data quality control and expression quantification. **(A)** Identification of recovered cells. The abscissa is the number of barcode sequences, and the ordinate is the number of UMIs. The barcode corresponding to the green line is the effective cell, and the gray line is the background noise. **(B, C)** Distribution of basic information (the number of genes, the total number of UMI and mitochondrial gene expression percentage) of each sample cell before and after filtering. **(B)** Distribution of basic information of each sample cell before filtering; **(C)** Distribution of basic information of each sample cell after filtering. **(D–G)** Scatter plot of basic information of each sample cell before and after filtering. **(D, F)** scatter plot before filtering. **(E, G)** scatter plot after filtering. **(D, E)** Relationship between nUMI and nGene. **(F, G)** Relationship between nUMI and pMito. The X-axis is the number of UMIs, and the Y-axis is the number of genes/mitochondria of mitochondria. The numbers above the graph are Pearson correlation coefficients for the number of uMI and the number of genes of mitochondria.

Supplementary Figure 2 Statistics on the number and percentage of different hemocyte clusters in cluster analysis.

Supplementary Figure 3 Expression profiles of TOP20 genes in each hemocytes cluster. **(A)** The heatmap of 20 up-regulated expression genes from four cell populations as a marker gene. **(B)** The dot plot of 20 up-regulated expression genes from four cell populations as a marker gene.

Supplementary Figure 4 Expression profiles of marker genes in each hemocytes cluster. **(A)** The heatmap of three marker genes from four cell clusters. **(B)** Expression pattern of the marker genes across cell clusters.

Supplementary Figure 5 GO functional annotation and KEGG pathway enrichment analysis of the differential genes among the hemocyte clusters. **(A)** Cluster 0/TGase^+^ cluster, **(B)** Cluster 1/CTL^+^ cluster, **(C)** Cluster 2/Crustin^+^ cluster, **(D)** Cluster 3/non-hemocytes. (A1-D1) Biological processes in GO functional annotation of the differential genes of each cluster. (A2-D2) Molecular functions in GO functional annotation of the differential genes of each cluster.

Supplementary Figure 6 Expression profiles of cell differentiation-related genes in each hemocytes cluster. **(A)** Heatmap showing the expression of genes related to the NOTCH signaling pathway among various clusters. **(B–D**) Differential expression of genes related to cell differentiation among cell clusters.

Supplementary Figure 7 Statistics on the number and percentage of different sub-clusters in each hemocyte cluster. **(A)** Cluster 0/TGase^+^ cluster, **(B)** Cluster 1/CTL^+^ cluster, **(C)** Cluster 2/Crustin^+^ cluster

Supplementary Figure 8 Identification of specific cell sub-clusters. **(A)** TGase^+^ cluster. **(B)** CTL^+^ cluster. **(C)** Crustin^+^ cluster.

Supplementary Figure 9 Schematic showing the functional characteristics of hemocytes morphological subpopulations, and their potential lineage flow based on hemocyte cluster profiles. **(A)** Schematic plot shows the functional characteristics of hemocytes morphological subpopulations. **(B)** The potential lineage flow of hemocytes morphological subpopulations. **(C)** The potential lineage flow of hemocytes morphological subpopulations based on hemocyte sub-cluster profiles.

Supplementary File 1 Top 20 marker genes for each cell type cluster. Shown are lists of the top 20 genes that show marker gene-like characteristics for each cell type cluster.

Supplementary File 2 GO functional annotation and KEGG pathway enrichment analysis of the differential genes among the hemocyte clusters.

Supplementary File 3 Differential expression profiles of key functional genes in various clusters.

Supplementary File 4 Expression profiles of cell differentiation-related genes in each hemocytes cluster.

Supplementary File 5 Expression profiles of genes for identifying the specific cell sub-clusters in each hemocytes cluster.

Supplementary File 6 Source data for CT values of each gene in different hemocyte subpopulations.
