# Supplementary figures and images for: Single-cell RNA-seq uncovered hemocyte functional subtypes and their differentiational characteristics and connectivity with morphological subpopulations in Litopenaeus vannamei
Source: Front Immunol. 2022 Sep 13;13:980021. doi: 10.3389/fimmu.2022.980021 (PMC9513592; doi:10.3389/fimmu.2022.980021)

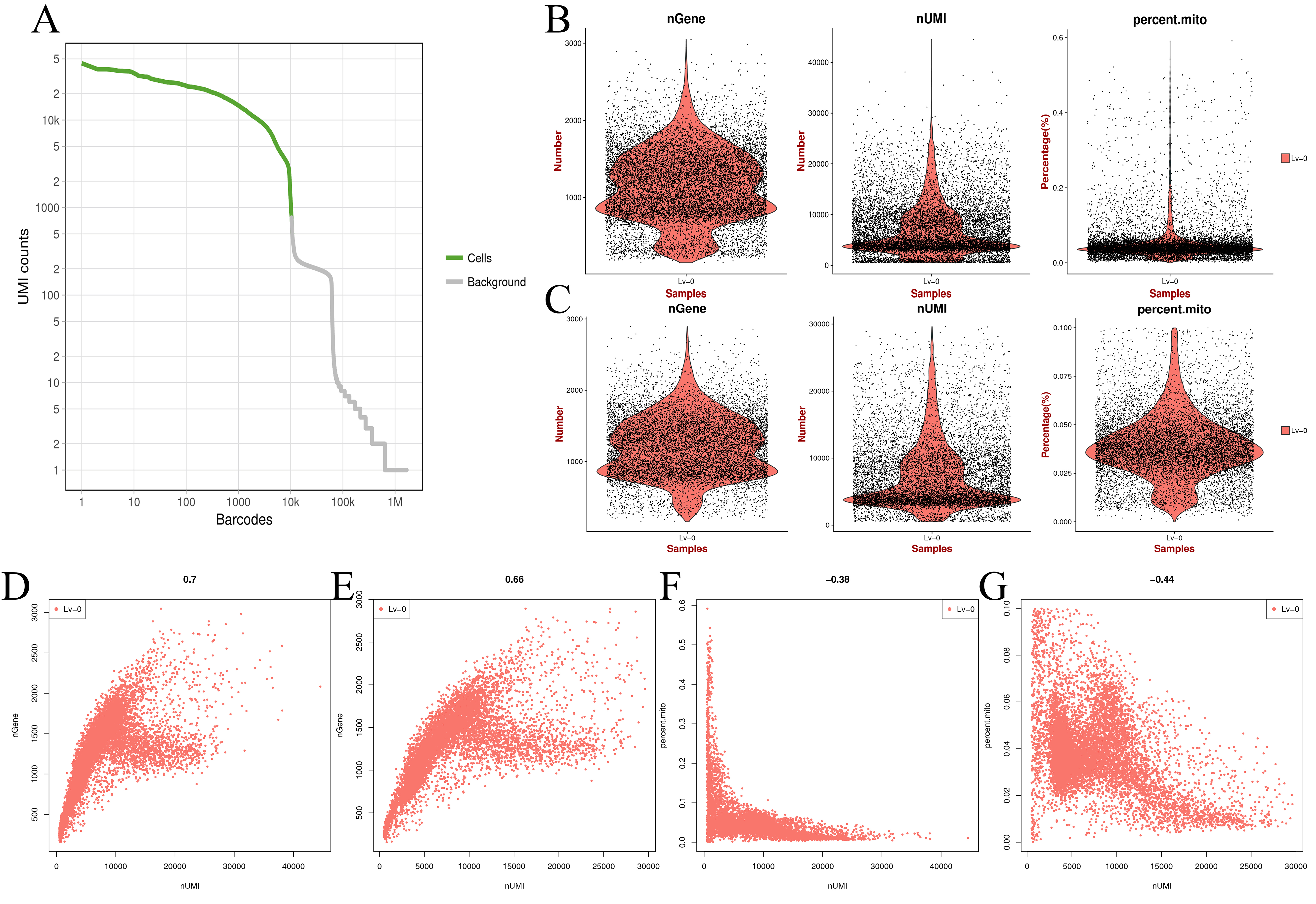

Supplement: Supplementary Figure 1 — Sequencing data quality control and expression quantification. (A) Identification of recovered cells. The abscissa is the number of barcode sequences, and the ordinate is the number of UMIs. The barcode corresponding to the green line is the effective cell, and the gray line is the background noise. (B, C) Distribution of basic information (the number of genes, the total number of UMI and mitochondrial gene expression percentage) of each sample cell before and after filtering. (B) Distribution of basic information of each sample cell before filtering; (C) Distribution of basic information of each sample cell after filtering. (D–G) Scatter plot of basic information of each sample cell before and after filtering. (D, F) scatter plot before filtering. (E, G) scatter plot after filtering. (D, E) Relationship between nUMI and nGene. (F, G) Relationship between nUMI and pMito. The X-axis is the number of UMIs, and the Y-axis is the number of genes/mitochondria of mitochondria. The numbers above the graph are Pearson correlation coefficients for the number of uMI and the number of genes of mitochondria. [file Image_1.jpeg]

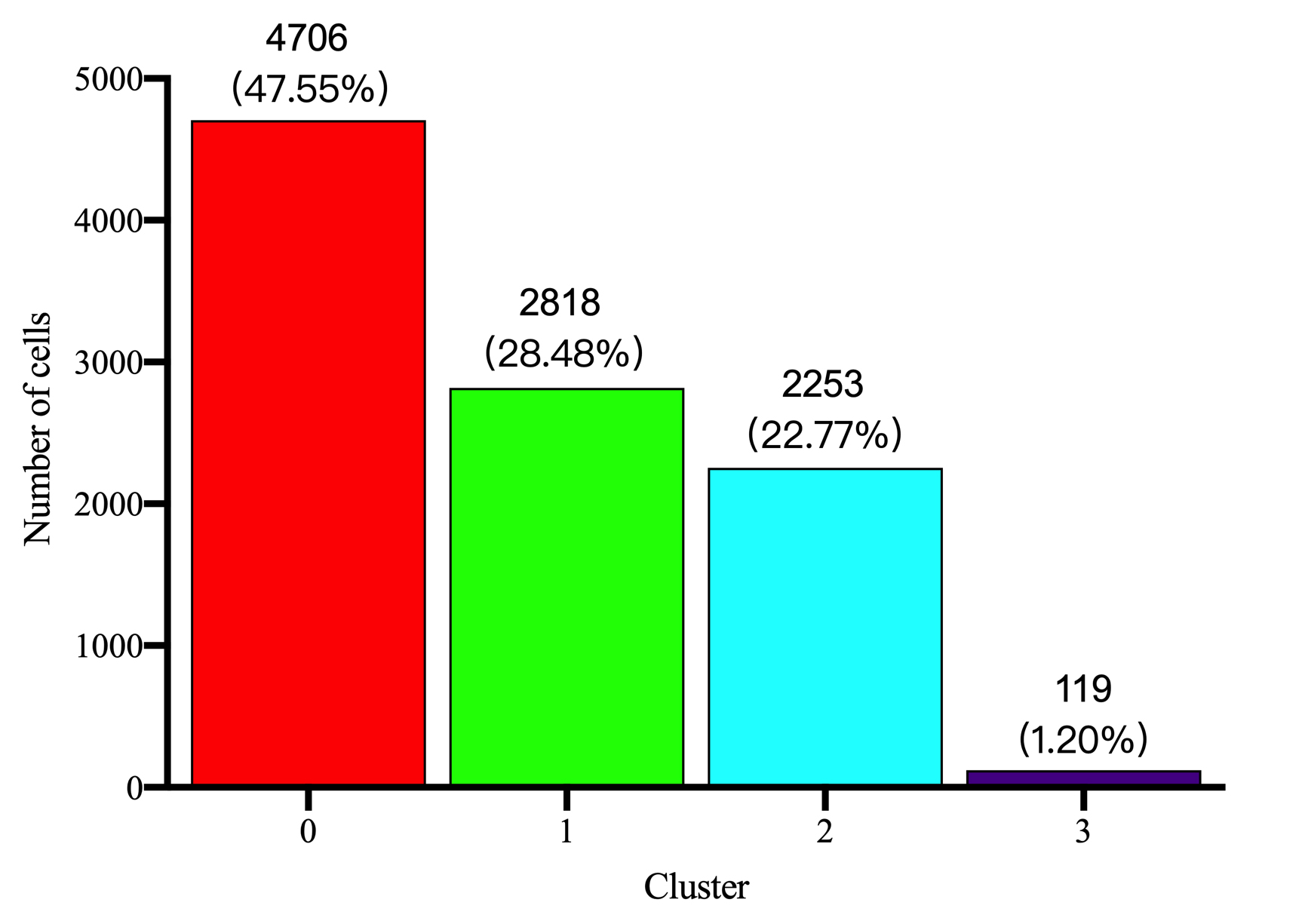

Supplement: Supplementary Figure 2 — Statistics on the number and percentage of different hemocyte clusters in cluster analysis. [file Image_2.jpeg]

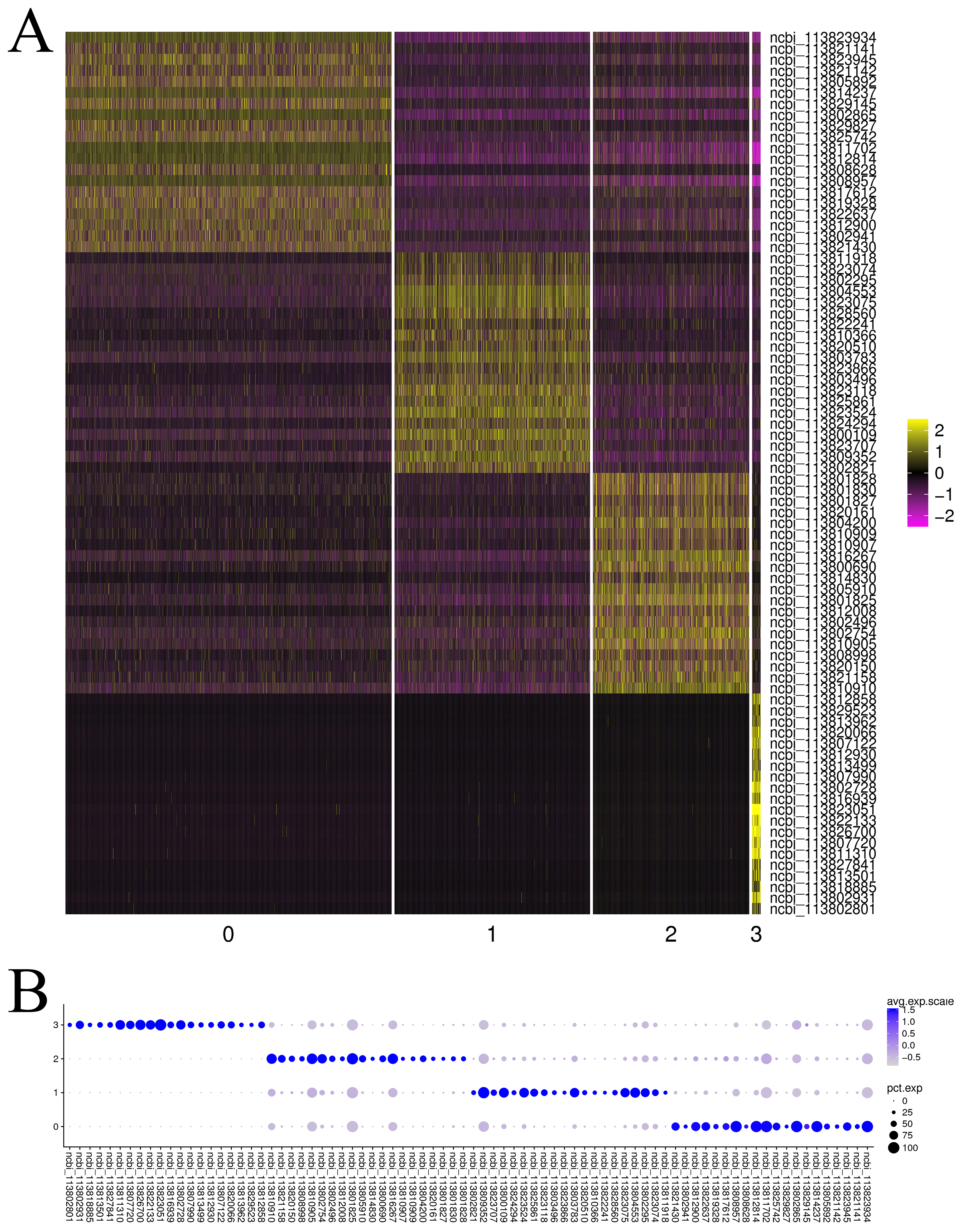

Supplement: Supplementary Figure 3 — Expression profiles of TOP20 genes in each hemocytes cluster. (A) The heatmap of 20 up-regulated expression genes from four cell populations as a marker gene. (B) The dot plot of 20 up-regulated expression genes from four cell populations as a marker gene. [file Image_3.jpeg]

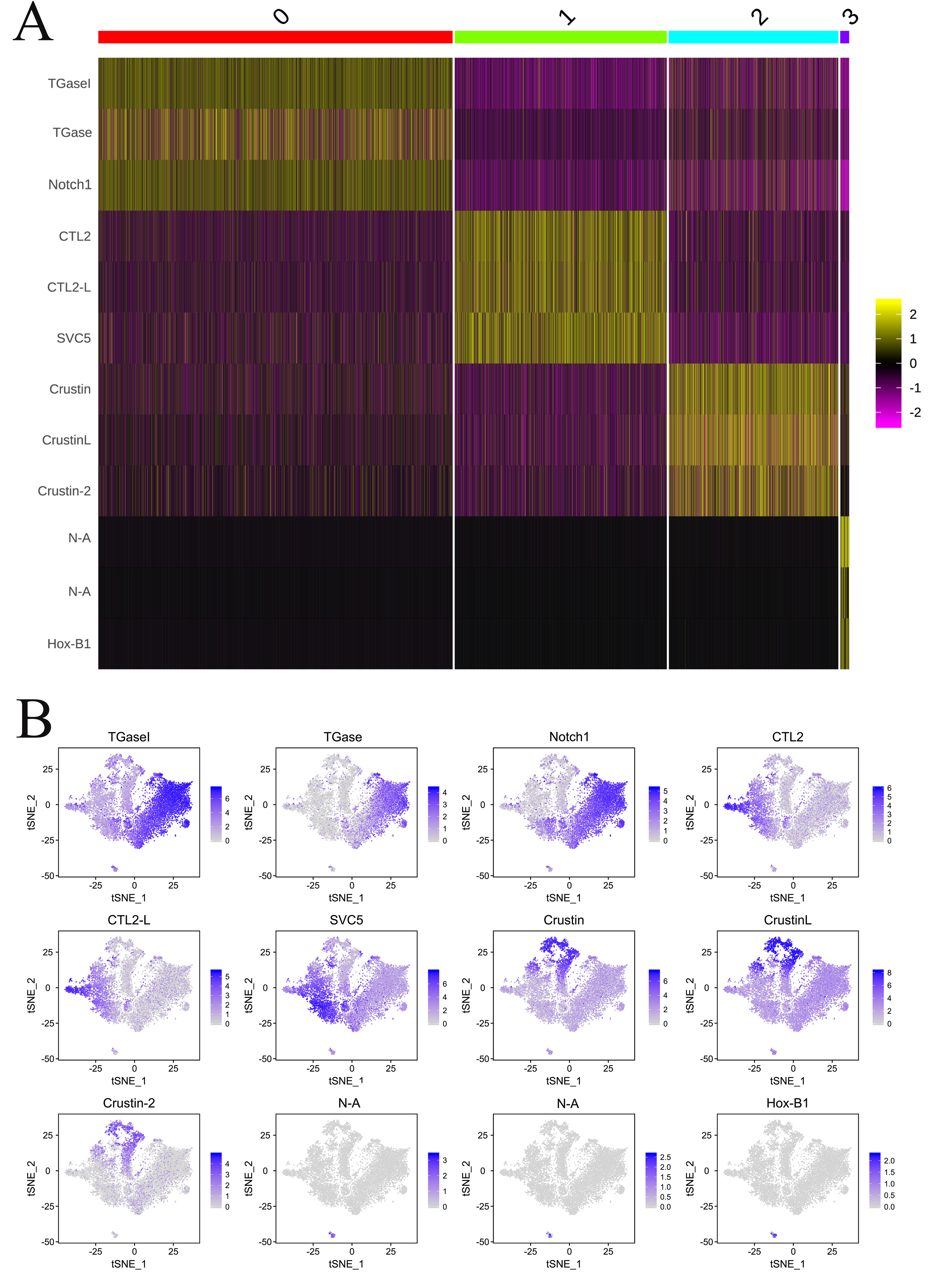

Supplement: Supplementary Figure 4 — Expression profiles of marker genes in each hemocytes cluster. (A) The heatmap of three marker genes from four cell clusters. (B) Expression pattern of the marker genes across cell clusters. [file Image_4.jpeg]

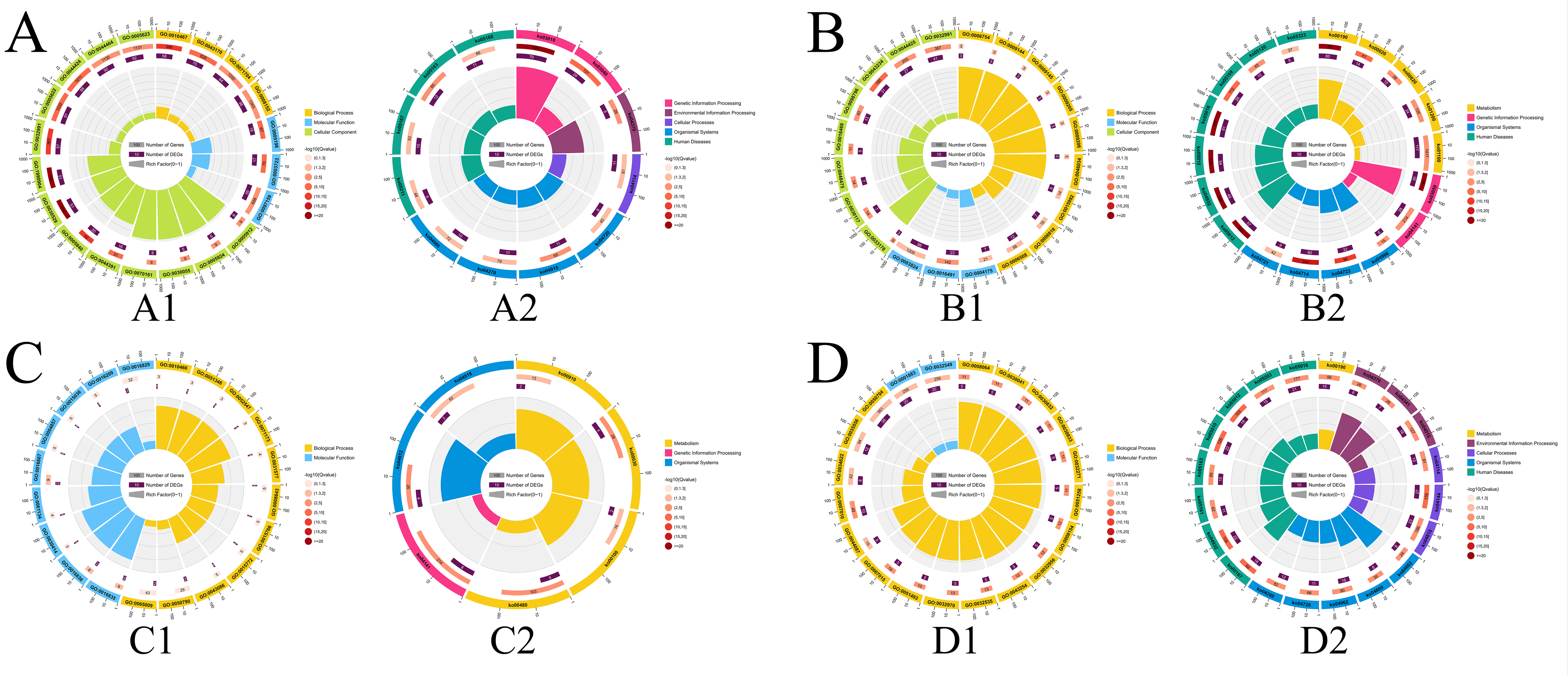

Supplement: Supplementary Figure 5 — GO functional annotation and KEGG pathway enrichment analysis of the differential genes among the hemocyte clusters. (A) Cluster 0/TGase+ cluster, (B) Cluster 1/CTL+ cluster, (C) Cluster 2/Crustin+ cluster, (D) Cluster 3/non-hemocytes. (A1-D1) Biological processes in GO functional annotation of the differential genes of each cluster. (A2-D2) Molecular functions in GO functional annotation of the differential genes of each cluster. [file Image_5.jpeg]

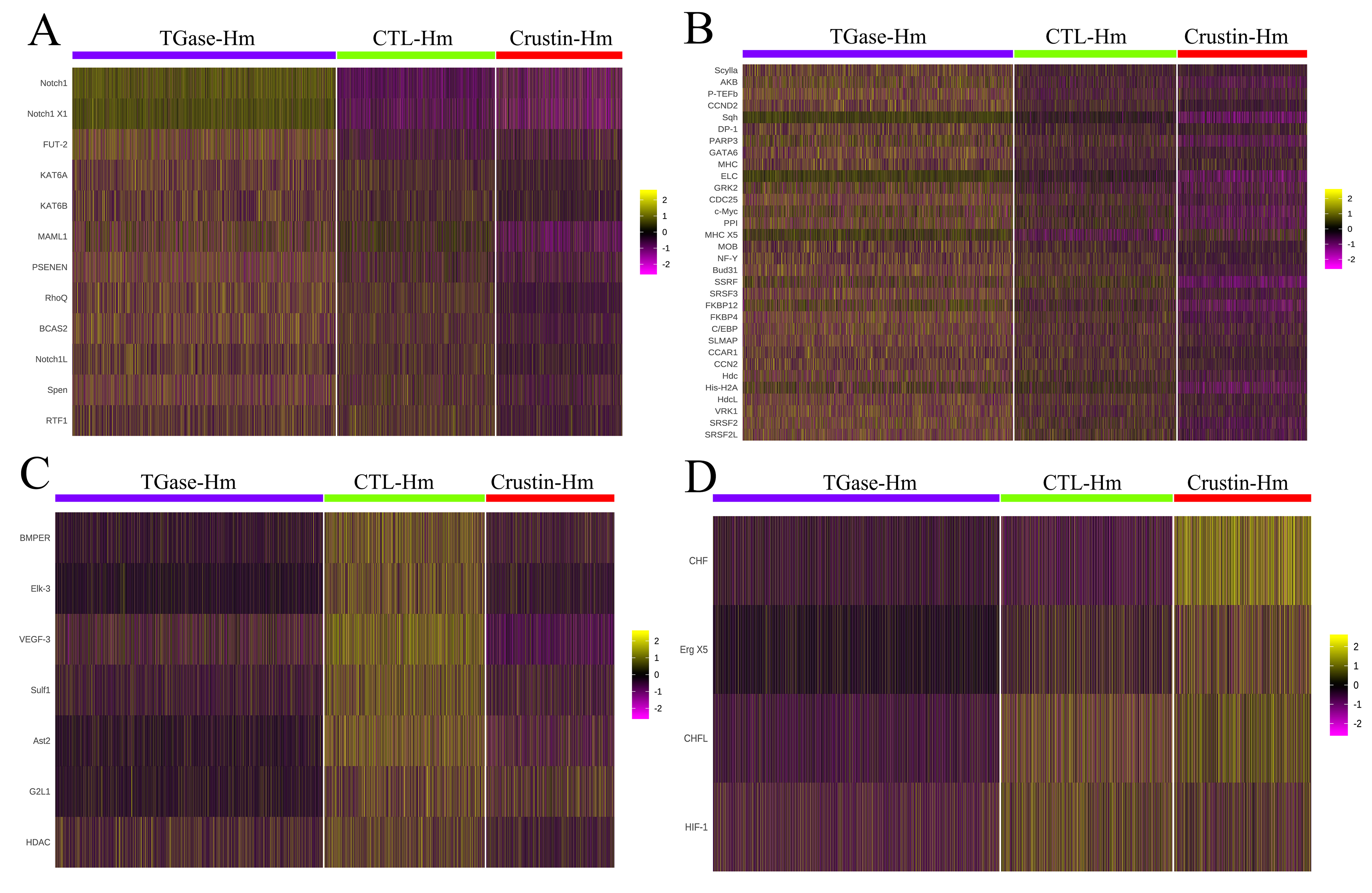

Supplement: Supplementary Figure 6 — Expression profiles of cell differentiation-related genes in each hemocytes cluster. (A) Heatmap showing the expression of genes related to the NOTCH signaling pathway among various clusters. (B–D) Differential expression of genes related to cell differentiation among cell clusters. [file Image_6.jpeg]

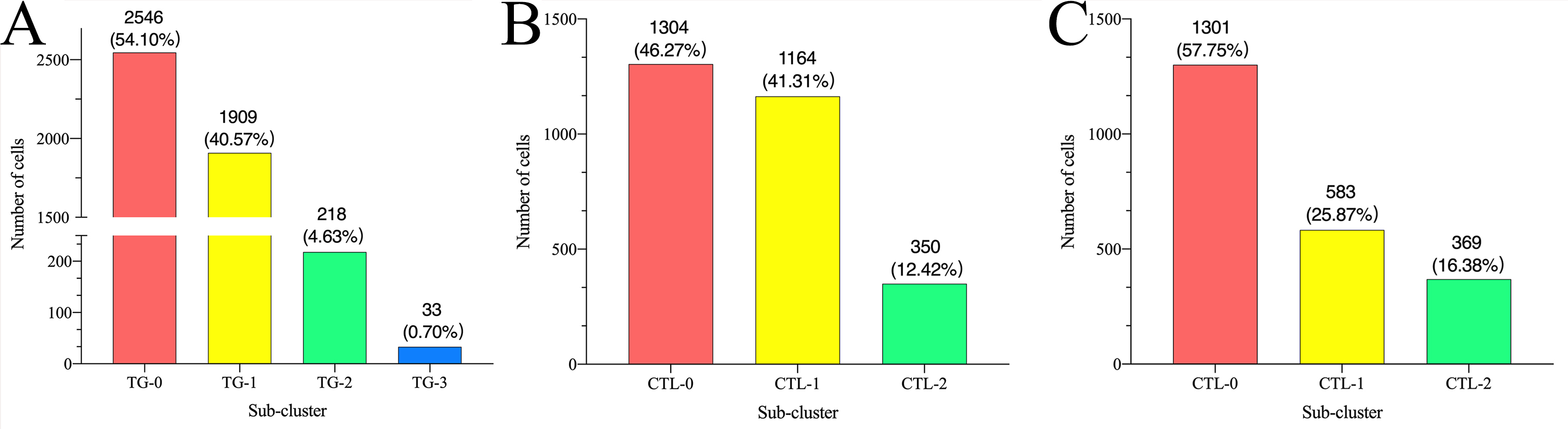

Supplement: Supplementary Figure 7 — Statistics on the number and percentage of different sub-clusters in each hemocyte cluster. (A) Cluster 0/TGase+ cluster, (B) Cluster 1/CTL+ cluster, (C) Cluster 2/Crustin+ cluster [file Image_7.jpeg]

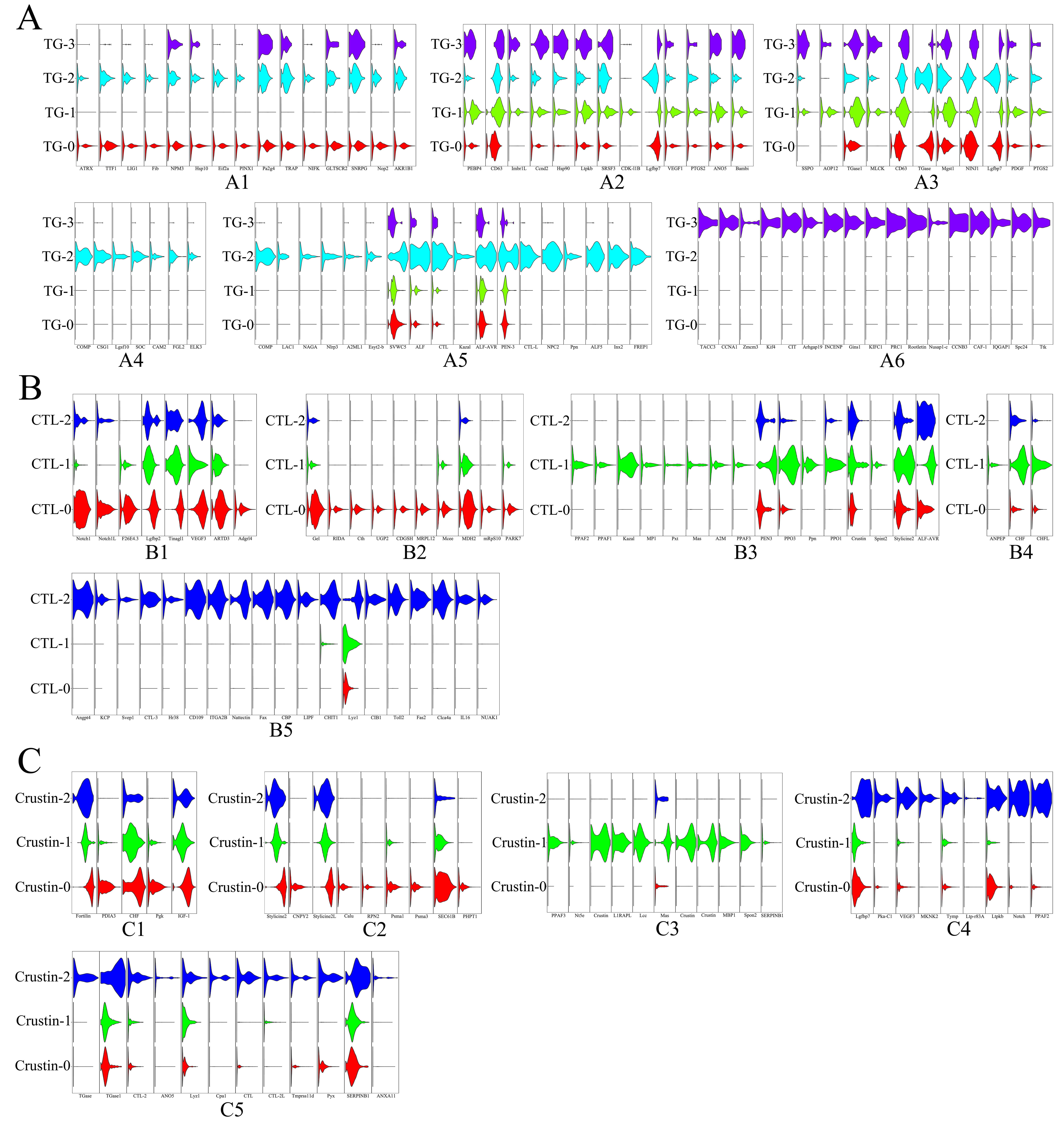

Supplement: Supplementary Figure 8 — Identification of specific cell sub-clusters. (A) TGase+ cluster. (B) CTL+ cluster. (C) Crustin+ cluster. [file Image_8.jpeg]

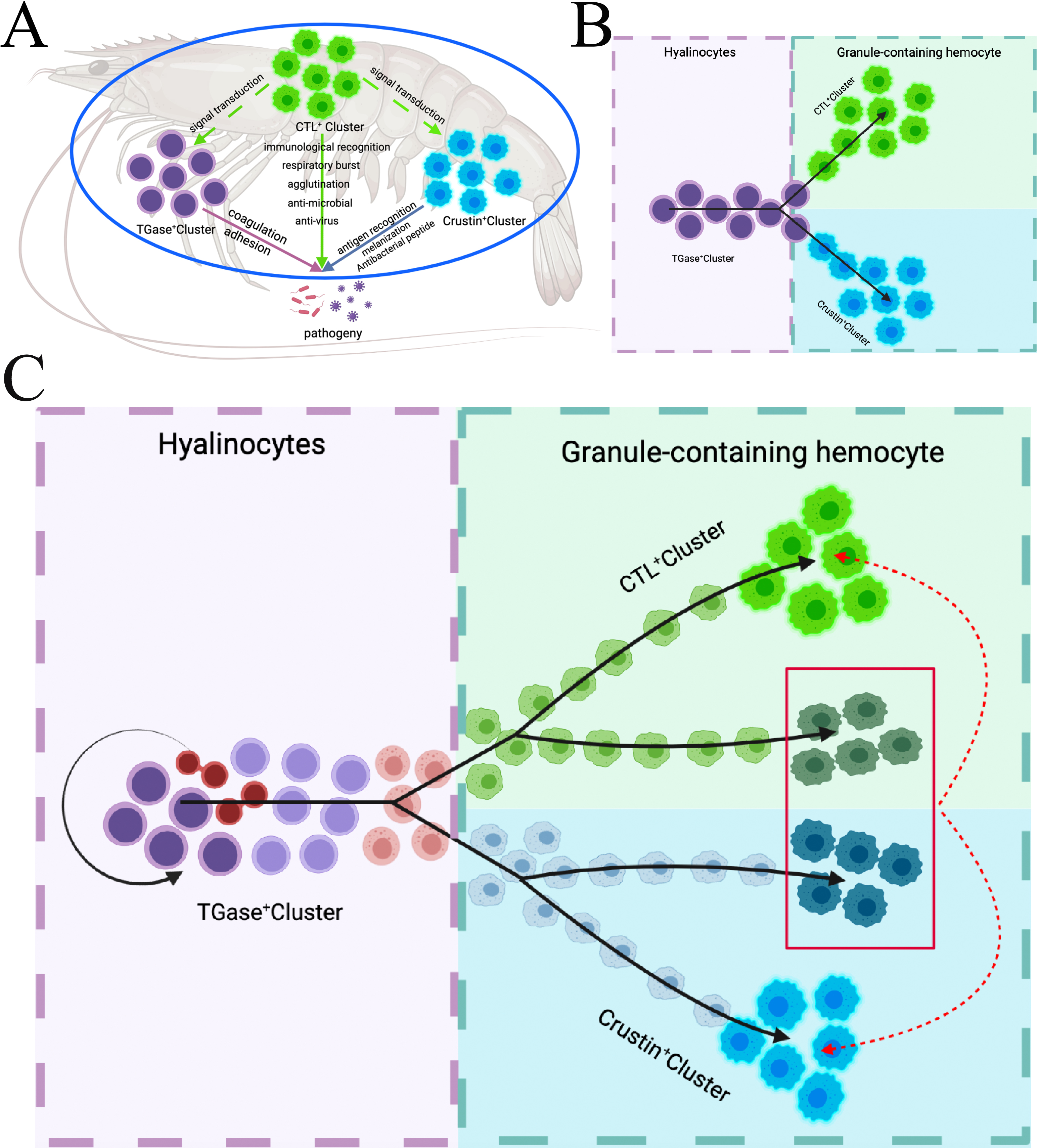

Supplement: Supplementary Figure 9 — Schematic showing the functional characteristics of hemocytes morphological subpopulations, and their potential lineage flow based on hemocyte cluster profiles. (A) Schematic plot shows the functional characteristics of hemocytes morphological subpopulations. (B) The potential lineage flow of hemocytes morphological subpopulations. (C) The potential lineage flow of hemocytes morphological subpopulations based on hemocyte sub-cluster profiles. [file Image_9.jpeg]
